# IPO5 promotes the proliferation and tumourigenicity of colorectal cancer cells by mediating RASAL2 nuclear transportation

**DOI:** 10.1186/s13046-019-1290-0

**Published:** 2019-07-09

**Authors:** Wenjuan Zhang, Yanxia Lu, Xiaomin Li, Jianming Zhang, Weihao Lin, Wei Zhang, Lin Zheng, Xuenong Li

**Affiliations:** 1 Department of Pathology, School of Basic Medical Sciences, Southern Medical University, Guangzhou, 510515 People’s Republic of China; 2 Department of General Surgery, Nanfang Hospital, Southern Medical University, Guangzhou, China

**Keywords:** Colorectal cancer, IPO5, RASAL2, NLS, Ras pathway, Karyopherins

## Abstract

**Background:**

Karyopherin nuclear transport receptors play important roles in tumour development and drug resistance and have been reported as potential biomarkers and therapeutic targets for tumour treatment. However, IPO5, one of the karyopherin nuclear transport receptor family members, remains largely uncharacterized in tumour progression.

**Methods:**

The TCGA data, quantitative reverse transcription-PCR (qRT-PCR), western blotting, and IHC analyses were used to detect IPO5 expression in CRC tissues. A series of in vivo and in vitro experiments was utilized to demonstrate the function of IPO5 in CRC tissues. Mass spectrometry (MS), CO-IP technology, subcellular fractionation, and immunofluorescence were utilized to investigate the possible mechanisms of CRC.

**Results:**

IPO5 was highly expressed and positively correlated with the clinicopathological characteristics of colorectal cancer tissues. Functional experiments indicated that IPO5 could promote the development of CRC. Mechanistically, we screened RASAL2, one cargo of IPO5, and further confirmed that IPO5 bound to the NLS sequence of RASAL2, mediating RASAL2 nuclear translocation and inducing RAS signal activation, thereby promoting the progression of CRC.

**Conclusions:**

Together, our results indicate that IPO5 is overexpressed in colorectal cancer cells. By transporting RASAL2, IPO5 may play a crucial role in CRC.

**Electronic supplementary material:**

The online version of this article (10.1186/s13046-019-1290-0) contains supplementary material, which is available to authorized users.

## Background

Colorectal cancer has become a major threat to human health. Although a large number of studies have been performed on CRC, the concrete molecular mechanisms of the formation and metastasis of CRC has not been completely clear until recently. Therefore, an in-depth study of the molecular mechanisms of the occurrence and development of CRC and the identification of effective molecular markers among many factors is of great importance.

Karyopherins are key regulatory molecules of nuclear plasma transport and are the most classic cell transporter proteins; they include both importins and exportins [1, 2]. Transporter proteins that have molecular weights greater than 40 kDa transport a variety of molecules between the cytoplasm and nucleus through the nuclear pore complex; these include transcription factors, splicing factors and other proteins [3–5]. Nevertheless, the dysfunction of karyopherins may derail the transport activity and may cause the abnormal localization of oncogenic factors, thus leading to tumourigenesis [6–8]. The currently reported karyopherins related to tumours include KPNA2, XPO5, XPO1, and KPNA7 [9–11]. IPO5, a member of the karyopherin beta subunit, locates in the 13q32 chromosomal region and has been demonstrated to play a vital role in the translocation of various proteins. However, the role of IPO5 in cancer progression has not been well defined. In our previous research, we analysed the CRC gene expression profile data of GSE41258 and sifted out a number of differentially expressed genes in which the expression of IPO5 was continuously increasing with the increasing severity from normal tissues to stage I, stage II, stage III, stage IV, and liver metastasis tumours (Additional file 1: Figure S1A) [12]. Meanwhile, the high expression of IPO5, especially in CRC cells, was also confirmed in the TCGA database and Oncomine database (Additional file 1: Figure S1B). Therefore, we hypothesized that IPO5 may play a key role in the development of CRC.

RASAL2, a member of the RAS GTPase-activating protein family, plays a role in negatively regulating RAS activity by catalysing the hydrolysis of RAS-GTP to RAS-GDP. Therefore, it is a key regulator of the RAS signalling pathway and is involved in many cellular activities. RAS signalling is intimately associated with the proliferation and metastasis of CRC cells. RASAL2 was reported to exhibit pro-tumourigenic or anti-tumourigenic effects in different types of cancer. Its role in colorectal cancer remains controversial.

In this study, we determined that the expression level of IPO5 is significantly upregulated in CRC tissues. Functional assays revealed that IPO5 could promote CRC growth in vitro and in vivo, and the mechanism involved was with the mediation of RASAL2 nuclear translocation followed by the activation of the RAS signalling pathway. Our study provides a potential oncogenic role for IPO5 in CRC development.

## Methods

### Cell culture

Cell lines SW620, SW480, HCT116, LoVo, HT-29, LS 174 T, Caco-2, RKO were obtained from American Type Culture Collection (ATCC, Manassas, VA, USA) and cultured in RPMI 1640 medium (Gibco, Grand Island, NY, USA) with 5% FBS at 37 °C with 5% CO_2_ (Gibco, USA). FHC cells were cultured in DMEM medium (Gibco, Grand Island, NY, USA) with 20% FBS (Gibco, Grand Island, NY, USA) at 37 °C in a humidified atmosphere with 5% CO_2_.

### Clinical specimens and animals

Human colorectal cancer tissues specimens were collected from patients with general surgery in Nanfang Hospital, Southern Medical University (Guangzhou, China), with informed consent from all patients. The fresh surgically resected CRC tissues were immediately frozen in liquid nitrogen and were stored at − 80 °C until further use. The use of clinical materials for research purposes has been approved by the Southern Medical University Institutional Board (Guangzhou, China). Female, 4–5 weeks old BALB/C nude mice were purchased from the Animal Center of Guangdong Province. All nude mice were raised under SPF conditions.

### RNA extraction and real-time quantitative PCR

The total RNA was extracted according to the instructions of Trizol reagent (Takara), and RNA reverse transcription were performed using Takara Reversal Kit. Quantitative real-time PCR (qRT-PCR) was done using the SybrGreen Qpcr Mix (DBI Bioscience). Relative expression levels were detected on ABI PRISM7500 Sequence Detection System (Applied Biosystem) [13]. The primer sequences are listed in Additional file 6: Table S4.

### Immunohistochemistry

Immunohistochemistry staining and scoring were performed according to previous research [13]. IPO5 (Bioss, Beijing; #bs-17075R), Ki67 (zsgb-bio, Beijing; #ZA-0502) and RASAL2 (Bioss, Beijing; #bs-21160R) antibodies were used for immunostaining.

### Construction of plasmids and transfection

The lentiviral constructs expressing or repressing IPO5 were purchased from Genechem (Shanghai, China), and the IPO5 siRNA was synthesized by Ribobio (Guangzhou, China). The RASAL2 wild type and RASAL2-NLS mutant plasmids were purchased from GeneCopoeia (USA). Cell transfection was performed using Lipofectamine 3000 as described in the manufacturer’s protocol (Invitrogen, USA).

### Western blot assay

The total protein was extracted using a lysis buffer (KeyGen Biotech, Nanjing, China) and the protein concentration was determined by bicinchoninic acid quantification kit (KeyGen Biotech, Nanjing, China). The protein was separated with 10% SDS-PAGE, and transferred onto PVDF membrane. Specific primary antibody was added: anti-GAPDH (proteintech, USA, #10494–1-AP), anti-p21 (Abcam, Cambridge, USA, #ab109199), anti-p27 (Abcam, Cambridge, USA, #ab92741), anti-cyclinD1 (proteintech, USA, #60186–1-lg), anti-p53 (proteintech, USA, #60283–2-lg), anti-PARP (Cell Signaling, Beverly, MA, #9542), anti-p-PARP (Cell Signaling, Beverly, MA, #9548), anti-Caspase-3 (Cell Signaling, Beverly, MA, #9662), anti-p-Caspase-3 (Cell Signaling, Beverly, MA, #9664), anti-Akt (Cell Signaling, Beverly, MA, #9279), anti-p-Akt (Ser-473) (Cell Signaling, Beverly, MA, #4060), anti-Erk (Cell Signaling, Beverly, MA, #9102), anti-p-Erk1/2 (Cell Signaling, Beverly, MA, #4370), anti-Mek (Cell Signaling, Beverly, MA, #4694), anti-p-Mek (Cell Signaling, Beverly, MA, #3958), anti-CDK4 (proteintech, USA, #11026–1-AP), anti-CDK6 (proteintech, USA, #14052–1-AP), anti-myc (Abcam, Cambridge, USA, #ab32072), anti-bax (Abcam, Cambridge, USA, #ab32503), anti-bcl2 (Abcam, Cambridge, USA, #ab32124) and incubated at 4 °C overnight followed by incubation with their respective second antibodies. The bands were visualized using Pierce ECL Western Blotting Substrate (Thermo Scientific, USA).

### Nude mice tumorigenicity assay

Female BALB/c nude mice aged 4–5 weeks were used and all animal experimental protocols were reviewed and approved by the Animal Care and Use Committee of Southern Medical University. In briefly, 1 × 10 ^7^ cells suspended in PBS were injected subcutaneously in the back of nude mice (*n* = 7 per group). The diameter of the tumor was measured every 3–4 days, tumor volume was calculated (V = 1/2*length*width*height). After 4 weeks, the tumor was excised and fixed in 10% formalin, followed by haematoxylin–eosin (HE) staining.

### CCK-8 cell proliferation, colony formation and transwell assay

Cell proliferation, colony formation assay, transwell migration assays were performed as previous described [13].

### Flow cytometry

The Cell Cycle Detection Kit (KeyGEN, Nanjing) and Fluorescein isothiocyanate (FITC), Annexin V, propidium iodide Apoptosis Detection Kit (KeyGEN, Nanjing) were used according to the manufacturer’s instruction.

### Subcellular fractionation

The nuclear plasma separation kit was purchased from TransGen Biotech (Beijing) and was carried out according to the instructions. Western blotting was used to verify the extraction. GAPDH antibody (proteintech, USA, #10494–1-AP) was used as cytoplasmic internal reference and Lamin B antibody (proteintech, USA, #23498–1-AP) was used as nuclear internal reference.

### Co-immunoprecipitation

Appropriate volume of IPO5 and RASAL2 antibodies (Santa Cruz Biotechnology, USA; #sc-390,605) were added to the extracted cell lysate and the mixture was shaken slowly overnight at 4 °C. Subsequent steps were performed as previous described and proteins was detected by western blot [14].

### Immunofluorescence

Cells were fixed and permeabilized with 0.5% TritonX-100, followed by blocked with 1% BSA for 30 min at room temperature. Thereafter, the primary anti-IPO5 (Santa Cruz Biotechnology; #sc-55,527) and RASAL2 (Santa Cruz Biotechnology; #sc-390,605) were added and incubated at 4 °C overnight. The next steps are the same as previous studies [14].

### Statistical analysis

SPSS 20.0 software (IBM) was used for data analysis. All data were presented as mean ± SD, comparison between groups using one-way ANOVA or independent t-test. The relationships between IPO5 expression and clinical pathological parameters were determined by χ^2^ test. Relationships between IPO5 expression and RASAL2 nuclear location were analysed by χ^2^ test. *p < 0.05, **p < 0.01, ***p < 0.001 was considered statistically significant.

## Results

### IPO5 is upregulated in colorectal cancer tissues

**Fig. 1:**
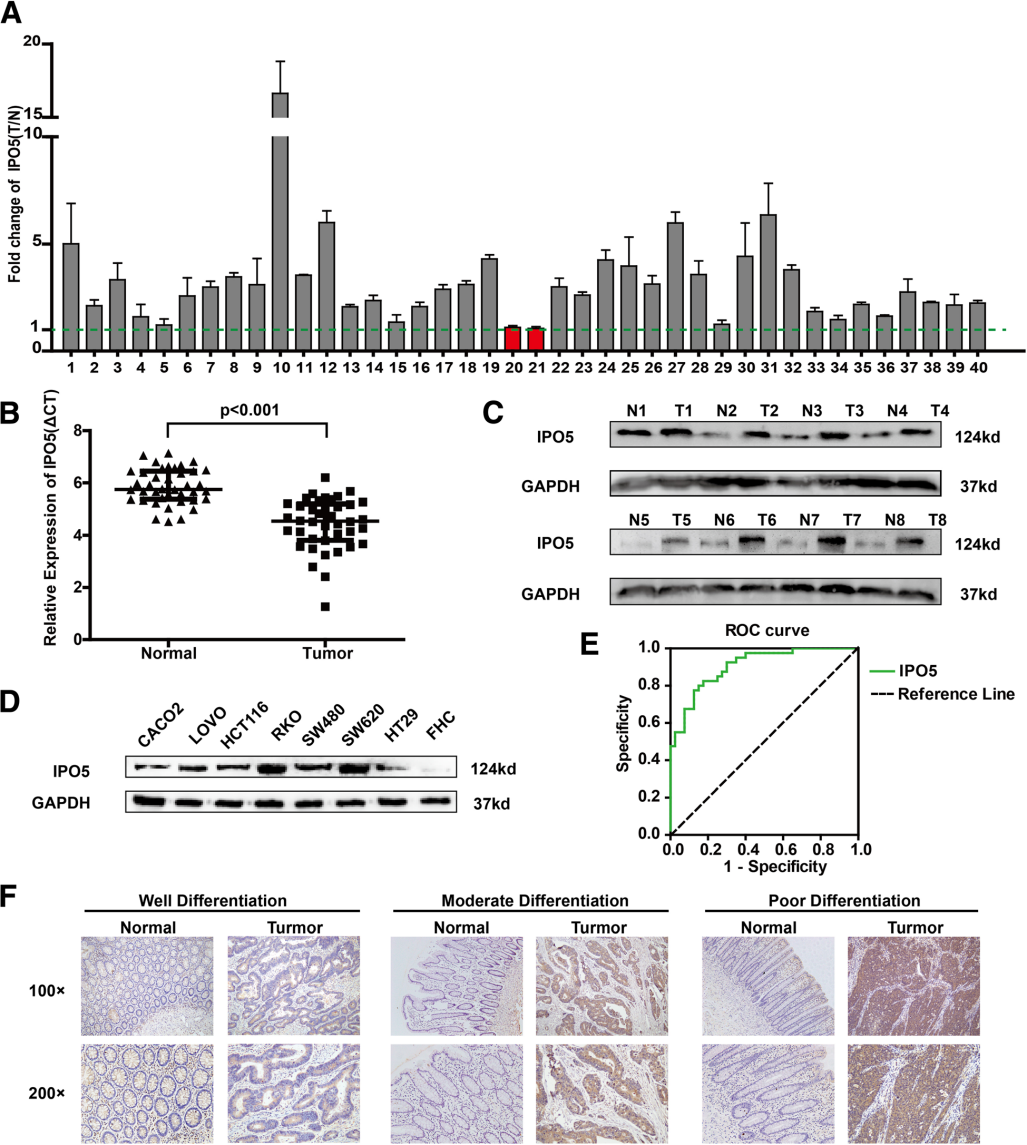
IPO5 was up-regulated in colorectal cancer tissues. **a** qRT-PCR analysis of IPO5 expression in 40 paired primary CRC tissues. Error bars represent the means ± SD of three independent experiments. **b** Comparison of IPO5 expression in 40 paired CRC tissues (T) and matched normal tissues (N) (*p* < 0.001). **c** Western blotting of IPO5 protein expression in 8 pairs of primary CRC (T) and adjacent normal intestine. **d** Western blotting of IPO5 expression between 8 CRC cell lines. **e** The ROC curve of IPO5. **f** Immunohistochemical staining (IHC) evaluation of the expression of IPO5 in paraffin-embedded human CRC tissues and adjacent normal tissues

The overexpression of IPO5 at the mRNA and protein levels was further verified using quantitative reverse transcription-PCR (qRT-PCR) and western blotting in 40 and 8 pairs of primary CRC and normal tissues, respectively (Fig. 1a-c). By evaluating the expression levels of IPO5 in eight colorectal cancer cell lines, LOVO, FHC, CACO2, RKO, SW620, SW480, HT29 and HCT116, we found that compared with that in the normal epithelial cell line FHC, IPO5 is highly expressed in cancer cells, and the highest expression levels were found in SW620, a cell line with high metastatic potential (Fig. 1d). To further investigate the potential clinical significance of IPO5 in CRC, we constructed ROC curves, using the relative expression levels of IPO5 in CRC tissues and paired non-cancerous tissues (AUC of 0.9050) (Fig. 1e). The data indicate IPO5 has potential as a biomarker for CRC diagnosis. Furthermore, the immunohistochemistry results showed that IPO5 is located predominantly in the cytoplasm and the positive staining of IPO5 was significantly higher in the cancer tissues than it was in the corresponding normal tissues (Fig. 1f). To further evaluate the relationship between the expression levels of IPO5 and the clinicopathological parameters, the expression of IPO5 was divided into a low-level group (*n* = 47) and a high-level group (*n* = 53). The results showed that the positive protein expression of IPO5 was positively correlated with tumour size, differentiation, TNM stage, lymph node metastasis (Table 1).

**Table 1:** Clinicopathologic characteristics of IPO5 expression in CRC patients

Clinicopathological Variables All cases	N 100	High expression 53	Low Expression 47	*X* ^*2*^	*P*
Age (years)					
≤ 60	48	26	22	0.050	0.49
> 60	52	27	25		0
Gender					
Male	56	28	28	0.460	
Female	44	25	19		0.549
Tumor size (cm)					
≤ 4.75	49	21	28	3.968	
> 4.75	51	32	19		0.036
Differentiation					
Well	20	7	13	7.647	
Moderate	61	30	31		0.020
Poor	19	16	3		
Serosal invasion					
Yes	40	20	20	0.241	
No	60	33	27		0.387
Lymph metastasis					
Yes	45	30	15	10.389	
No	55	23	32		0.006
TNM classification					
I–II	65	29	36	10.585	
III–IV	35	24	11		0.014

### Knockdown of IPO5 inhibits the growth and migration of CRC cells in vitro and in vivo

**Fig. 2:**
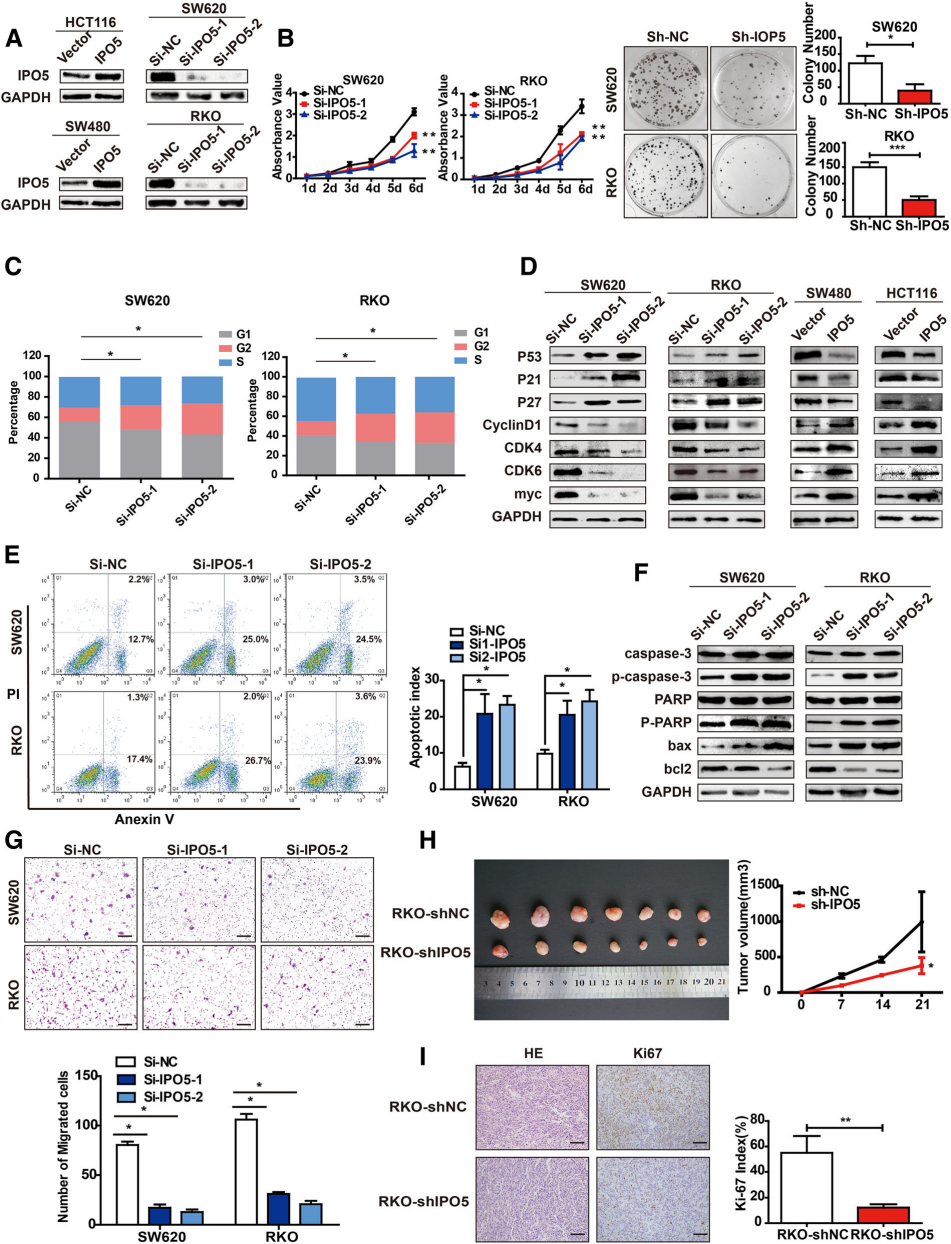
Knockdown of IPO5 inhibits the growth and migration of colorectal cancer cells. **a** Overexpression or knockdown efficiency of IPO5 was confirmed by western blotting. **b** IPO5 knockdown significantly inhibited cell growth on the basis of CCK8 and colony formation assay. Error bars represent the means ± SD of 5 or 3 independent experiments. ***p < 0.001. **c** Cell cycle was detected by flow cytometry after silence IPO5 expression. **d** The expression levels of Cycle-related proteins were detected by western blotting. **e** Knockdown of IPO5 increased apoptotic cells as analyzed by flow cytometry. **f** Expression of apoptosis protein markers were analyzed using western blotting. **g** Silencing of IPO5 reduced CRC cells migration was showed by transwell assays. Error bars represent the means ± SD of 5 different fields. *p < 0.05. **h** IPO5 knockdown inhibited growth of RKO xenografts in nude mice (*n* = 7). Right panel showed the tumor sizes change. The data were calculated as the mean tumor volumes ± SD for 7 samples. **i** Representative images of IHC staining and Ki67-positive Sections of subcutaneous tumors. Error bars represent the means ± SD of 5 different fields

To characterize the biological role of IPO5 in CRC cells, we overexpressed IPO5 in the SW480 and HCT116 cell lines, simultaneously silenced IPO5 expression with IPO5-shRNA in SW620 and RKO cells (Fig. 2a), and then selected IPO5-shRNA1 to construct lentiviral stable interfering IPO5 cell lines for subsequent functional studies. The upregulation of IPO5 significantly increased the activity (*P* < 0.01) and clonogenicity (P < 0.01) of HCT116 and SW480 cells compared to those of the normal control cells (Additional file 2: Figure S2A and S2B). Conversely, interference with IPO5 reduces cell viability (P < 0.01) and clonal formation (P < 0.01) (Fig. 2b). The cell cycle distribution was detected by flow cytometry. The results showed that the overexpression of IPO5 increased the proportion of G1 phase cells and decreased the proportion of G2/M phase cells (Additional file 2: Figure S2C). In contrast, the silencing of IPO5 resulted in the opposite result (Fig. 2c). Therefore, IPO5 can induce G2-M phase transition in CRC cells. We used western blotting to detect the effects of IPO5 on cell cycle-related proteins; the results showed that the overexpression of IPO5 significantly upregulated the expression of CyclinD1, CDK4, CDK6 and Myc and downregulated the expression of p53, p21, p27 and the opposite results were obtained after interference with IPO5 (Fig. 2d). We then examined the effects of IPO5 on the apoptosis of CRC cells induced by 5-fluorouracil. The results showed that IPO5 depletion sensitized CRC cells to 5-fluorouracil treatment as detected by increased apoptotic rates (*P* < 0.01) (Fig. 2e) and elevated apoptotic marker expression levels (Fig. 2f). Thus, we estimate that IPO5 may be associated with 5-fluorouracil resistance in CRC. Next, the effect of IPO5 on cell motility was measured by a transwell assay. The ectopic expression of IPO5 significantly promoted cell migration compared to that in the control cells (*P* < 0.05) (Additional file 2: Figure S2D). The opposite results were obtained in cells with the repression of IPO5 (P < 0.01) (Fig. 2g). To confirm the tumourigenic ability of IPO5 in vivo, RKO-shNC and RKO-shIPO5 cells were subcutaneously injected into nude mice. As shown in the figure, IPO5 down-regulation markedly reduced the size of RKO xenografts in nude mice (Fig. 2h). Coincidently, fewer proliferating cells were detected in RKO-shIPO5 xenografts, as indicated by the Ki-67 assay (P < 0.01) (Fig. 2i).

### Upregulation of IPO5 correlates with 5-fluorouracil resistance in CRC cells

**Fig. 3:**
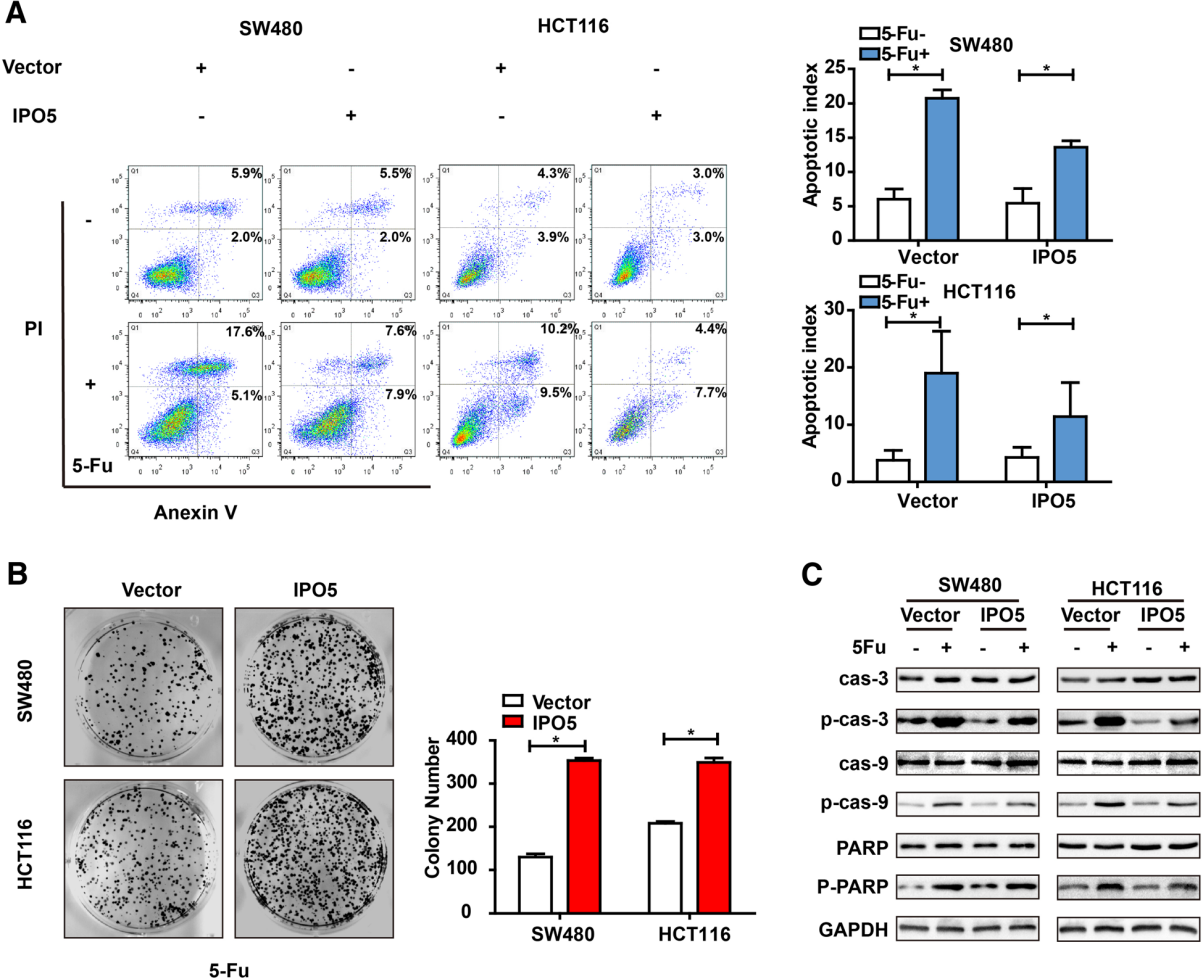
Upregulation of IPO5 correlates with 5-fluorouracil resistance in CRC cells **a** Stably expressing IPO5 or the control CRC cells were treated with 5-fluorouracil for indicated time and analyzed cell apoptotic proportion by flow cytometry. **b** Effect of IPO5 overexpression on 5-fluorouracil resistance in SW480 and HCT116 cells was detected by colony formation assay. **c** The corresponding CRC cells treated with or without 5-fluorouracil were subjected to immunoblot analysis using apoptotic protein markers

To further verify that IPO5 is associated with 5-fluorouracil resistance in colorectal cancer cells, we examined the effect of IPO5 overexpression on apoptosis induced by 5-FU. The results showed that cells with upregulated IPO5 expression were less sensitive towards 5-FU-induced apoptosis compared with that of the control cells (Fig. 3a). Consistent with the apoptosis results, the colony formation in IPO5-overexpressing CRC cells was significantly higher than that in the mock cells in response to 5-fluorouracil treatment (Fig. 3b). Treatment with 5-fluorouracil increased the levels of the apoptotic markers cleaved caspase-3, and cleaved PARP. However, the upregulation of IPO5 partially reduced this effect (Fig. 3c). These results indicate that the expression of IPO5 might contribute to acquired drug resistance to 5-fluorouracil in CRC cells.

### RASAL2 represents a novel IPO5 cargo protein

**Fig. 4:**
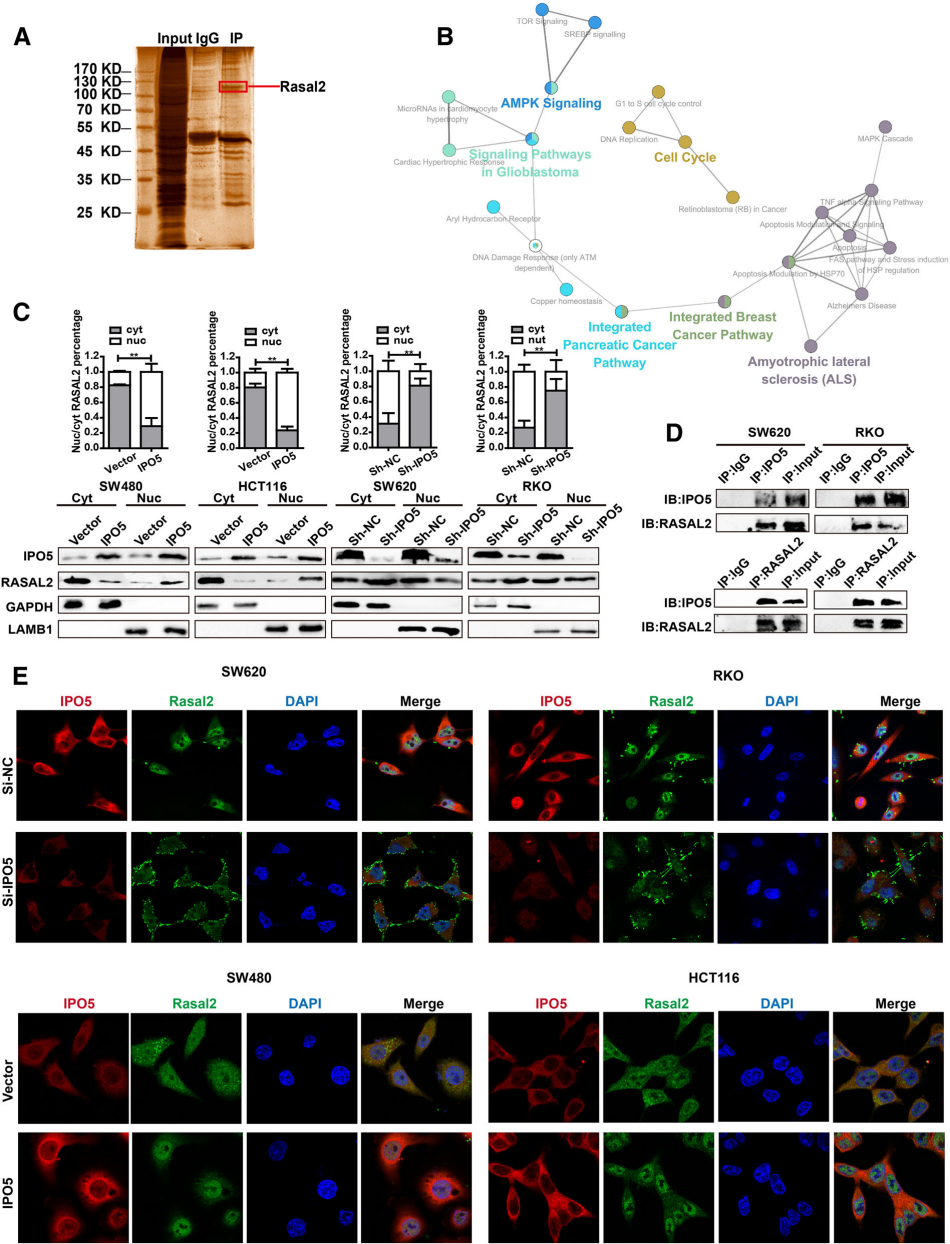
RASAL2 represent novel IPO5 cargo protein **a** CO-IP technology was used to identify IPO5 interaction proteins. After silver staining, specific protein bands were collected and then identified by mass spectrometry (MS). **b** Evaluation of the biological processes of the putative IPO5 binding partners. Candidates were conducted on the pathway analysis with Cytoscape softwar and Wikipathway database. **c** Subcellular fractionation was performed to illustrate that RASAL2 is one of the cargos of IPO5. Data represent the mean ± SD of 3 independent experiments. *P < 0.05. **d** CO-IP assays was used to further validation the interaction between RASAL2 and IPO5. **e** Representative fluorescence images of the location change of RASAL2 in the cells knockdown or overexpress IPO5

To explore the molecular mechanisms of IPO5-mediated regulation in CRC cells, we applied CO-IP technology followed by mass spectrometry (MS) to identify the proteins interacting with IPO5 (Fig. 4a). After removing the common false-positive hits, a total of 640 proteins were identified (Additional file 3: Table S1). To clarify the specific biological processes involving IPO5, 640 proteins were further examined using DAVID combined with KEGG pathway enrichment analysis. We found that in addition to the known functionality in the database, there are also notable features of interest, such as cell cycle regulation and cytoskeletal regulation (Additional file 4: Table S2). In addition, we built biological networks and analysed the signal pathways involved in these 640 proteins by using Cytoscape software plug-in ClueGO in conjunction with the WikiPathway database, and we found that these proteins were involved in various cancer-related signal pathways (Fig. 4b). To further pinpoint potential proteins transported to the nucleus by IPO5, we used the cNLS Mapper Tool together with the COMPARTMENTS database to determine the nuclear localization signal and subcellular localization of the proteins. Finally, 15 proteins with NLS sequences and shuttles through the nucleoplasm were screened, which we believe are the most likely cargo candidates for IPO5 (Additional file 5: Table S3). From these 15 proteins, we focused on 8 cancer-related proteins (UBR5, ATRX, RASAL2, LIMK1, RAD51, RABL6, SIN3A, and DNAJB1) for further subcellular fractionation analysis. The results showed that RASAL2 was the most likely cargo protein for IPO5 in CRC cells (Additional file 2: Figure. S2E). To verify our results, we continued to explore whether the subcellular localization of RASAL2 was affected by IPO5. The results showed that the amounts of cytoplasmic RASAL2 increased together with a concomitant decrease in the nuclear levels after silencing IPO5, whereas cells overexpressing IPO5 showed the opposite results (Fig. 4c). Furthermore, we performed an endogenous reciprocal CO-IP assay to verify the interaction of IPO5 and RASAL2 (Fig. 4d). Then, immunofluorescence and confocal microscopy were used to demonstrate that RASAL2 translocated into the nucleus in IPO5 upregulated cells but accumulated in the cytoplasm and at the cell surface in si-IPO5-treated cells (Fig. 4e). These results collectively suggest that IPO5 mediates RASAL2 nuclear transport in CRC cells.

### IPO5 mediates RASAL2 nuclear transport by NLS

**Fig. 5:**
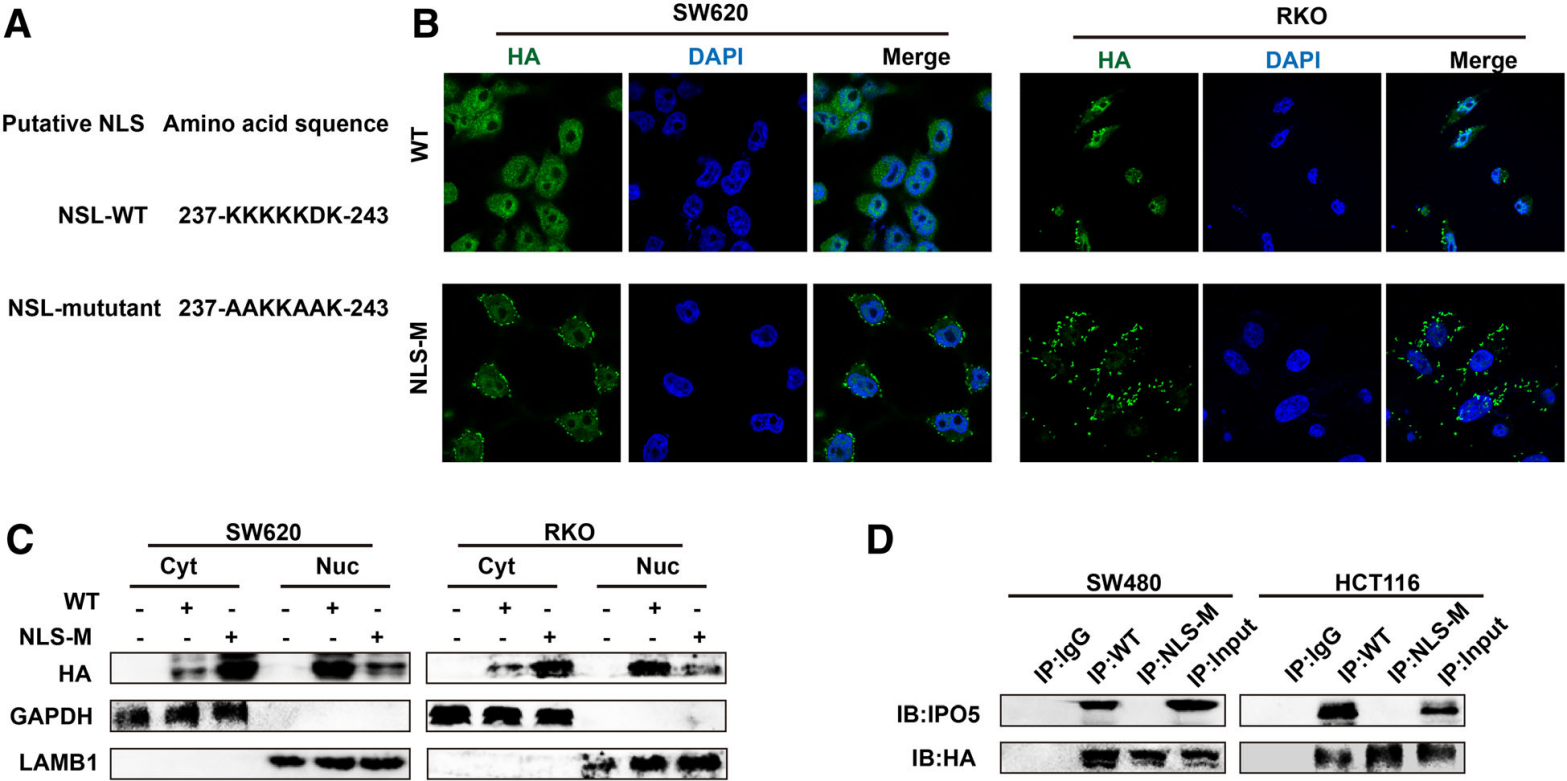
IPO5 mediates RASAL2 nuclear transport by NLS **a** Putative NLS sequence segments of RASAL2 protein. **b** Immunofluorescence images exhibit the localizations of the exogenously expressed RASAL2 wild type and putative NLS mutant fused with HA in CRC cells. **c** The effect of WT and putative NLS mutant plasmid on the distribution of RASAL2 was tested by subcellular fractionation method in CRC cells. **d** The impact of NLS mutant on the interaction between RASAL2 and IPO5 was detected by CO-IP assays

As we already know, IPO5 usually transports cargo into the nucleus by NLS dependency. Therefore, we analysed the RASAL2 amino acid sequence and identified putative NLSs based on NLS software. The sequence was used to construct a point mutation plasmid fused with HA fluorescent protein to perform subsequent experiments (Fig. 5a). As shown, the RASAL2-WT protein exhibited nuclear and cytoplasmic localization. However, the NLS mutant showed mainly cytoplasmic and dispersed membrane fluorescence (Fig. 5b). A similar result was also validated by immunoblot analyses (Fig. 5c). Further, the CO-IP assay demonstrated that the RASAL2-NLS mutant protein could not co-precipitate IPO5 compared with the co-precipitation ability of the wild-type NLS protein (Fig. 5d). These results suggest that the NLS sequence (residues 237–243) of RASAL2 was required for IPO5-mediated RASAL2 nuclear transport.

### IPO5-mediated nuclear import of RASAL2 is required for RAS pathway activation and CRC development

**Fig. 6:**
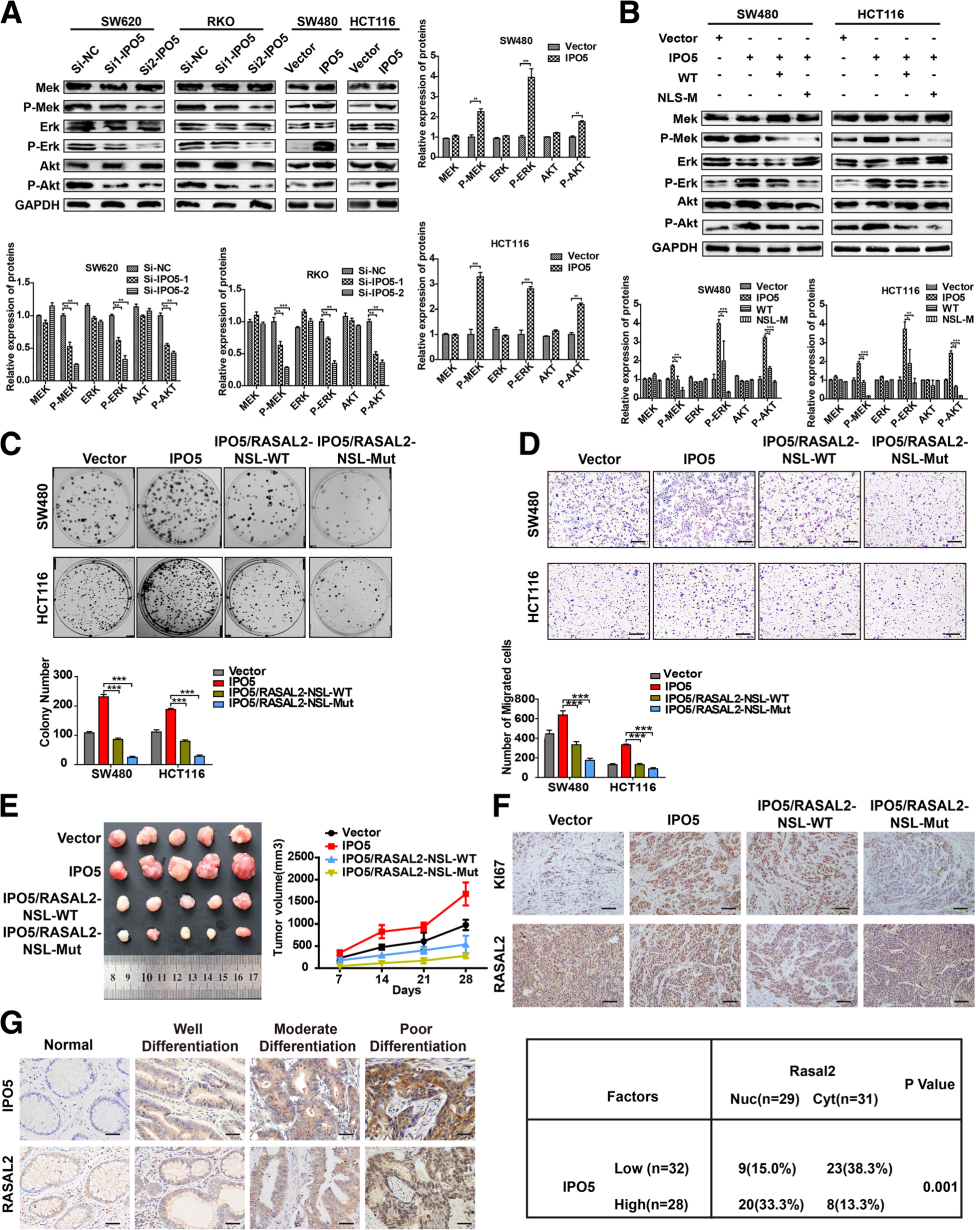
IPO5-mediated nuclear import of RASAL2 is required for RAS pathway activation and CRC development **a** Western blotting was used to determine the expression of ERK, p-ERK, AKT, and p-AKT upon knockdown or overexpressing of IPO5 in CRC cells. **b** The NLS mutant of RASAL2 resulted in reversing the IPO5-mediated RAS signaling activation showed by Western blotting. **c** and **d** The recovery effect of NLS mutant on the IPO5-mediated CRC cells migration and proliferation ability were detected by colony formation and transwell respectively. Scale bars, 200 μm. **e** Subcutaneous tumorigenesis in nude mice was applied to explore the impact of NLS mutant on tumorigenicity of IPO5 in vivo. Data represent the mean ± SD of 3 independent experiments. *P < 0.05. **f** Tumor xenograft tissues were embedded in paraffin and then assessed for the expression of Ki-67 and RASAL2. Scale bars, 200 μm. **g** Correlation of nuclear RASAL2 expression and IPO5 expression in colorectal cancer tissue was evaluated by Immunohistochemical. Scale bars, 50 μm

Given the major role of RASAL2 in regulating the RAS pathway, we investigated whether IPO5 could modulate the RAS pathway. Strikingly, western blotting showed that interference with IPO5 caused a significant decrease in p-Erk, p-Mek, and p-Akt. However, the overexpression of IPO5 causes an increase in these proteins (Fig. 6a). To further characterize whether RASAL2 contributes to this phenotype, we transfected the RASAL2-WT and RASAL2-NLS-MUT plasmids into HCT116 and SW480 cells that overexpressed IPO5, respectively, and performed a western blot analysis. The results showed that RASAL2-NLS-MUT can significantly abolish IPO5-mediated RAS signalling activation and the expression of RAS target proteins, while RASAL2-WT can only partially perform these functions (Fig. 6b). Similar to this observation, compared to that of RASAL2-NLS-WT, RASAL2-MUT more obviously abrogated the tumour-promoting function of IPO5 in vitro and in vivo, as indicated by colony formation, transwell experiments and subcutaneous tumourigenicity (Fig. 6c, d and e). Furthermore, we performed immunohistochemical staining on subcutaneous tissues to examine the relationships between nuclear RASAL2, Ki-67 and IPO5. We found that compared with that of the RASAL2-NLS-WT group, RASAL2-NLS-MUT significantly reversed RASAL2 nuclear accumulation induced by IPO5 overexpression (Fig. 6f). Similarly, by analysing several serial sections from 60 CRC patients, we found that the RASAL2 protein was detected in the nucleus mainly in high IPO5 expression regions, while RASAL2 was restricted to cytoplasmic distribution in the low IPO5 expression sections (*P* < 0.01) (Fig. 6g). Finally, we concluded that IPO5-mediated RASAL2 nuclear transport is required for CRC progression.

## Discussion

It has been well established that the dysfunction of karyopherins can lead to the abnormal localization of oncogenes and tumour suppressor genes and contributes to the uncontrolled growth and drug resistance of cancer cells [15, 16]. The frequently observed aberrant expression of karyopherins has been reported to be involved in multiple tumours [17–19]. For example, CRM1, KPNB1 and KPNA2 are overexpressed in cervical cancer cells [20, 21]. KPNA2 associates with glioblastomas and colon cancer [22, 23]. The abnormal expression of CRM1 is associated with acute myeloid leukaemia and oesophageal carcinoma [24, 25]. KPNA4 is responsible for prostate cancer metastasis [26]. IPO5 belongs to the karyopherin protein family and usually transports RNA or protein into the nucleus by binding to the nuclear localization signal (NLS) sequence. However, unlike the other karyopherin members, IPO5 has been rarely reported in tumours. Until now, existing studies have shown that IPO5 can mediate the nuclear translocation of the HPV-16 E5 oncoprotein, a virus-associated protein that may be involved in cervical carcinogenesis [27]. Additionally, IPO5 mRNA was reported to be a target of miRNA produced by human herpesvirus 8, a process that may be associated with Kaposi’s sarcoma [28]. Additionally, IPO5 mediates the transport of histones H3, H4, and the ribosomal protein L7 and participates in ribosome synthesis [29]. Moreover, IPO5 can bind to SMAD1 to regulate BMP signalling [30]. These findings, together with our previous database analysis, suggest that IPO5 may play a role in oncogenesis. To our knowledge, this report is the first to elaborate that the altered expression of IPO5 is accompanied by the progression of CRC.

Since preceding studies have shown that the carcinogenic role of karyopherins is attributed to the abnormal transport of cargo proteins [31–33], we hypothesized that the increased nuclear import of IPO5 cargo proteins may be the cause of the observed oncogene phenotype. Here, CO-IP technology followed by mass spectrometry was utilized to search for IPO5 cargo proteins in CRC cells. Many proteins detected have been previously shown to interact with IPO5. The global evaluation of the biological processes of the putative IPO5 cargo revealed several biological pathways, such as viral cell cycle and mRNA metabolic process, which agree with the historical data. This indicates some credibility of the mass spectrometry results, which may facilitate our search for the cargo proteins of IPO5. Then, by analysing the 640 proteins from the mass spectrometry data using the cNLS mapper tool [34, 35] and the COMPARTMENTS database coupled with subsequent subcellular fractionation methods, we found that RASAL2 may be one of the cargos transported by IPO5 in CRC cells.

RASAL2 is a RAS-GTPase-activated protein and a negative regulator of the RAS cascade. Its potential oncosuppressor role was currently hypothesized in a variety of tumours by inhibiting the activation of the downstream RAS pathway [36, 37]. Existing studies have observed that RASAL2 is mainly localized and plays a role in the cytoplasm [38, 39], but whether it could enter the nucleus has not been reported. Here, via immunofluorescence and immunohistochemical staining combined with protein localization database results, we found that RASAL2 is distributed not only in the cytoplasm but also in the nucleus and membranes of CRC cells. This led us to analyse its amino acid sequence by using a related database, and we found that it contains one NLS sequence, which indicates that RASAL2 is very likely to enter the nucleus with the NLS sequence. Therefore, we hypothesized that IPO5 may interact with the NLS of RASAL2, resulting in RASAL2 nuclear translocation and eliminating the inhibitory effect of RASAL2 in the cytoplasm; this would subsequently augment the RAS pathway and would ultimately drive CRC progression. To validate this hypothesis, we constructed a RASAL2 NLS sequence plasmid with point mutations and transfected cells for further functional experiments. Our results show that the NLS mutations of RASAL2 disrupted the interaction between IPO5 and RASAL2 and significantly impaired the IPO5-mediated malignant characteristics of CRC. There is evidence that some karyopherins share common cargo [40, 41], and this is coupled with the fact that our exploration for the IPO5 binding cargo still lacks specificity; therefore, we must bear in mind that is a possibility that RASAL2 enters the nucleus, which is also mediated by other IPO5 family members. These results invite us to carry out further studies. Because altered karyopherin protein functions play a vital role in drug resistance [42, 43], recent studies point to their potential usefulness as a novel strategy for anticancer therapy [44]. A small molecule inhibitor targeting CRM1 has been shown to be beneficial to the anticancer effects [45–47]. INI-43, an inhibitor of KPNB1, is a potent chemotropic agent of malignancies [48]. However, because normal cells also share the nuclear transport machinery with cancer cells, drugs targeting these proteins are currently limited. Therefore, prospective basic research with regard to the tumourigenic mechanisms of karyopherin proteins is lacking and is desperately required for effective cancer therapy.

## Conclusions

**Fig. 7:**
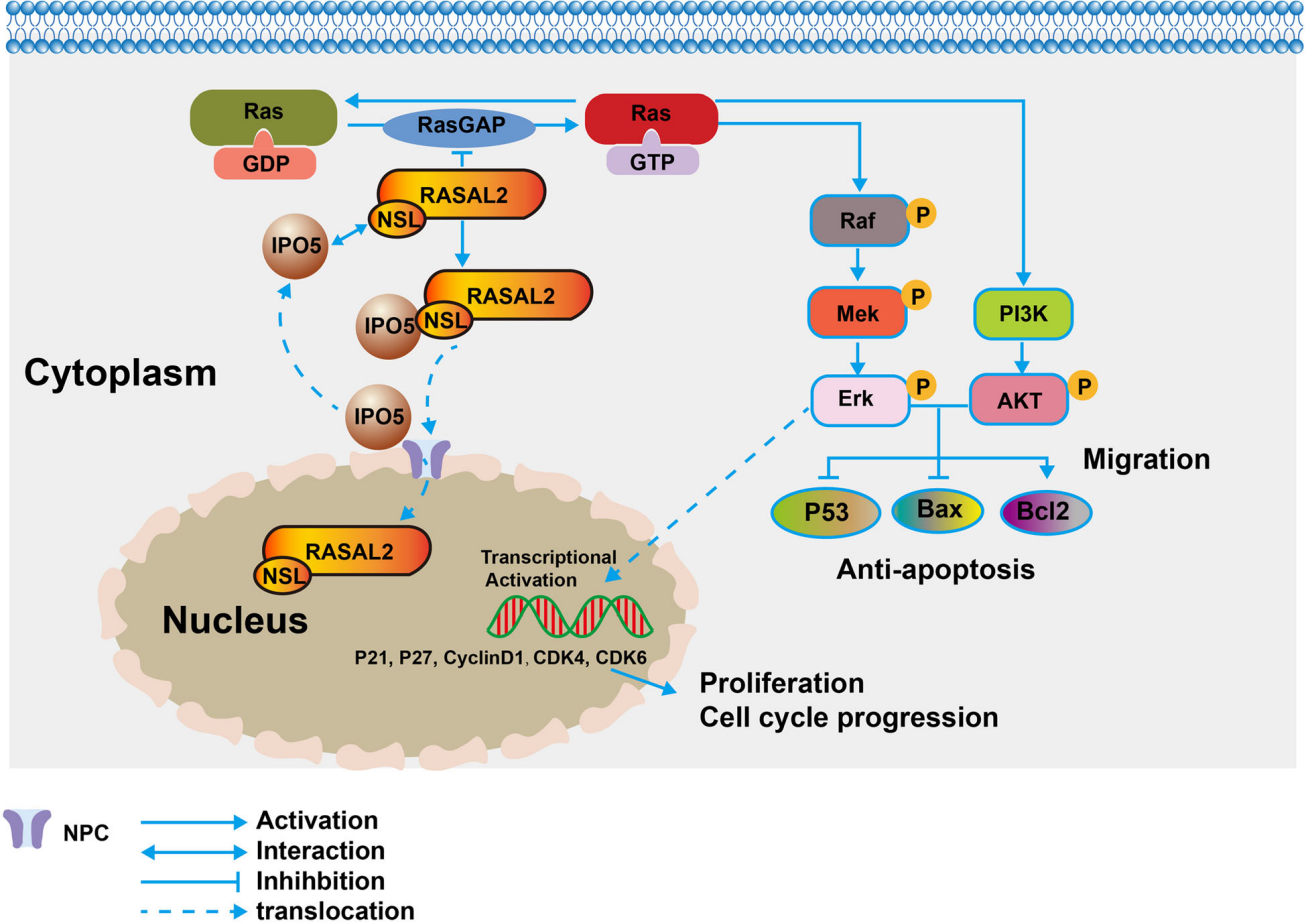
Illustration of IPO5 involvement in CRC. IPO5 interacts with the NLS sequence of RASAL2 leading to RASAL2 nuclear transposition, This preventing RASAL2 from functioning as a RAS signaling pathway suppressor in the cytoplasm. Thus resulting in RAS signal activation and CRC growth and metastasis

In summary, the current study demonstrated that IPO5 is an oncogene involved in CRC cell proliferation and migration. This highlights the significance of IPO5 in 5-fluorouracil-resistant CRC cells. The oncogenic function of IPO5 was mediated by promoting RAS signalling by increasing the nuclear translocation of RASAL2 (Fig. 7). Our results provide new information on the carcinogenic role mediated by IPO5. It is expected to be a promising therapeutic target.

## Additional files

Additional file 1: **Figure S1.** The expression of IPO5, data from public database. (A) Analysis of IPO5 expression in different types of malignancies. (B) Analysis of IPO5 expression in TCGA CRC large sample genomic database. (C) Analysis of IPO5 expression using the CRC gene expression profile data GSE41258. (JPG 849 kb)

Additional file 2: **Figure S2.** Effects of IPO5 over-expression on CRC cell proliferation and migration in vitro (A and B) Up-regulation of IPO5 increased cell proliferation (*P* < 0.01) and clonogenicity (P < 0.01) as compared to controls . (C) The effect of IPO5 on the cell cycle distribution was detected by flow cytometry in CRC cells. (D) The impact of ectopic expression of IPO5 on cell migration was validated by transwell assay(P < 0.01). (E) Screen out IPO5 transporting cargos using subcellular fractionation methods followed by immunoblotting. (JPG 5002 kb)

Additional file 3: **Table S1.** List of IPO5 binding candidates identified by mass spectrometry. (DOCX 55 kb)

Additional file 4: **Table S2.** Pathway analysis of IPO5 binding candidates using DAVID tool. (DOCX 16 kb)

Additional file 5: **Table S3.** List of 15 IPO5 binding proteins with NLS sequence. (DOCX 20 kb)

Additional file 6: **Table S4.** Primer sequences used in RT-qPCR analysis. **Table S5**: Nucleotide sequences used for knockdown. (DOC 40 kb)

## Data Availability

The datasets used and/or analysed during the current study are available from the corresponding author on reasonable request.

## Abbreviations

5-FU: 5-fluorouracil
BSA: Albumin from bovine serum
CCK-8: Cell counting kit-8
CO-IP: Co-Immunoprecipitation
CRC: Colorectal cancer
CRM1: Exportin CRM1
FBS: Fetal bovine serum
IF: Immunofluorescence
IHC: Immunohistochemistry
IPO5: Importin 5
MS: Mass spectrometry
NC: Negative control
NLS: Nuclear localization sequence
NPC: Nuclear pore complex
qPCR: Quantitative real-time polymerase chain reaction
RASAL2: RAS protein activator like 2
TCGA: The Cancer Genome Atlas
TNM: Tumor Node Metastasis

## References

[1] Chook YM, Blobel G (2001). Karyopherins and nuclear import. Curr Opin Struct Biol.

[2] Çağatay T, Chook YM (2018). Karyopherins in cancer. Curr Opin Cell Biol.

[3] Lund E, Guttinger S, Calado A, Dahlberg JE, Kutay U (2004). Nuclear export of microRNA precursors. Science.

[4] Silver PA, Kau TR, Way JC (2004). Nuclear transport and cancer: from mechanism to intervention. Nat Rev Cancer.

[5] Tran EJ, King MC, Corbett AH (2014). Macromolecular transport between the nucleus and the cytoplasm: advances in mechanism and emerging links to disease. Biochim Biophys Acta.

[6] Senapedis WT, Baloglu E, Landesman Y (2014). Clinical translation of nuclear export inhibitors in cancer. Semin Cancer Biol.

[7] Kimura M, Imamoto N (2014). Biological significance of the importin-beta family-dependent nucleocytoplasmic transport pathways. Traffic.

[8] Kosyna Friederike, Depping Reinhard (2018). Controlling the Gatekeeper: Therapeutic Targeting of Nuclear Transport. Cells.

[9] Shigeyasu K, Okugawa Y, Toden S, Boland CR, Goel A (2017). Exportin-5 functions as an oncogene and a potential therapeutic target in colorectal cancer. Clin Cancer Res.

[10] Kim J, McMillan E, Kim HS, Venkateswaran N, Makkar G, Rodriguez-Canales J (2016). XPO1-dependent nuclear export is a druggable vulnerability in KRAS-mutant lung cancer. Nature.

[11] Vuorinen EM, Rajala N, Rauhala HE, Kallioniemi A (2016). Abstract 76: KPNA7 nuclear import protein - a critical regulator of cancer cell growth. Cancer Res.

[12] Qi L, Ding Y (2018). Construction of key signal regulatory network in metastatic colorectal cancer. Oncotarget.

[13] Lin C, Zhang J, Lu Y, Li X, Zhang W, Zhang W, et al. NIT1 suppresses tumour proliferation by activating the TGFβ1–Smad2/3 signalling pathway in colorectal cancer. Cell Death Dis. 2018;9:263. 10.1038/s41419-018-0333-3 PMC5833788 29449642

[14] Zeng Z, Li Y, Pan Y, Lan X, Song F, Sun J, et al. Cancer-derived exosomal miR-25-3p promotes pre-metastatic niche formation by inducing vascular permeability and angiogenesis. Nat Commun. 2018;9:5395. 10.1038/s41467-018-07810-w PMC6300604 30568162

[15] Weis Karsten (2003). Regulating Access to the Genome. Cell.

[16] Beck M, Schirmacher P, Singer S (2017). Alterations of the nuclear transport system in hepatocellular carcinoma - new basis for therapeutic strategies. J Hepatol.

[17] Zhao Xingchen, Chen Yuanhan, Tan Xiaofan, Zhang Li, Zhang Hong, Li Zhilian, Liu Shuangxin, Li Ruizhao, Lin Ting, Liao Ruyi, Zhang Qianmei, Dong Wei, Shi Wei, Liang Xinling (2018). Advanced glycation end-products suppress autophagic flux in podocytes by activating mammalian target of rapamycin and inhibiting nuclear translocation of transcription factor EB. The Journal of Pathology.

[18] Yan Dongqing, Pomicter Anthony D., Tantravahi Srinivas, Mason Clinton C., Senina Anna V., Ahmann Jonathan M., Wang Qiang, Than Hein, Patel Ami B., Heaton William L., Eiring Anna M., Clair Phillip M., Gantz Kevin C., Redwine Hannah M., Swierczek Sabina I., Halverson Brayden J., Baloglu Erkan, Shacham Sharon, Khorashad Jamshid S., Kelley Todd W., Salama Mohamed E., Miles Rodney R., Boucher Kenneth M., Prchal Josef T., O'Hare Thomas, Deininger Michael W. (2018). Nuclear–Cytoplasmic Transport Is a Therapeutic Target in Myelofibrosis. Clinical Cancer Research.

[19] Kau TR, Silver PA (2003). Nuclear transport as a target for cell growth. Drug Discov Today.

[20] van der Watt PJ, Maske CP, Hendricks DT, Parker MI, Denny L, Govender D (2009). The Karyopherin proteins, Crm1 and Karyopherin β1, are overexpressed in cervical cancer and are critical for cancer cell survival and proliferation. Int J Cancer.

[21] Angus L, van der Watt PJ, Leaner VD (2014). Inhibition of the nuclear transporter, Kpnβ1, results in prolonged mitotic arrest and activation of the intrinsic apoptotic pathway in cervical cancer cells. Carcinogenesis.

[22] Zhang Y, Zhang M, Yu F, Lu S, Sun H, Tang H (2015). Karyopherin alpha 2 is a novel prognostic marker and a potential therapeutic target for colon cancer. J Exp Clin Cancer Res.

[23] Li J, Liu Q, Liu Z, Xia Q, Zhang Z, Zhang R (2018). KPNA2 promotes metabolic reprogramming in glioblastomas by regulation of c-myc. J Exp Clin Cancer Res.

[24] Kojima K, Kornblau SM, Ruvolo V, Dilip A, Duvvuri S, Davis RE (2013). Prognostic impact and targeting of CRM1 in acute myeloid leukemia. Blood.

[25] Yang X, Cheng L, Yao L, Ren H, Zhang S, Min X (2014). Involvement of chromosome region maintenance 1 (CRM1) in the formation and progression of esophageal squamous cell carcinoma. Med Oncol.

[26] Yang J, Lu C, Wei J, Guo Y, Liu W, Luo L (2017). Inhibition of KPNA4 attenuates prostate cancer metastasis. Oncogene.

[27] Krawczyk E, Hanover JA, Schlegel R, Suprynowicz FA (2008). Karyopherin beta3: a new cellular target for the HPV-16 E5 oncoprotein. Biochem Biophys Res Commun.

[28] Quan L, Qiu T, Liang J, Li M, Zhang Y, Tao K (2015). Identification of target genes regulated by KSHV miRNAs in KSHV-infected lymphoma cells. Pathol Oncol Res.

[29] Soniat M, Cagatay T, Chook YM (2016). Recognition elements in the histone H3 and H4 tails for seven different importins. J Biol Chem.

[30] Baas R, Sijm A, van Teeffelen HA, van Es R, Vos HR, Marc TH (2016). Quantitative proteomics of the SMAD (suppressor of mothers against decapentaplegic) transcription factor family identifies importin 5 as a bone morphogenic protein receptor SMAD-specific importin. J Biol Chem.

[31] Vuorinen EM, Rajala NK, Rauhala HE, Nurminen AT, Hytonen VP, Kallioniemi A (2017). Search for KPNA7 cargo proteins in human cells reveals MVP and ZNF414 as novel regulators of cancer cell growth. Biochim Biophys Acta Mol basis Dis.

[32] Wang CI, Chien KY, Wang CL, Liu HP, Cheng CC, Chang YS (2012). Quantitative proteomics reveals regulation of karyopherin subunit alpha-2 (KPNA2) and its potential novel cargo proteins in nonsmall cell lung cancer. Mol Cell Proteomics.

[33] Alshareeda AT, Negm OH, Green AR, Nolan CC, Tighe P, Albarakati N (2015). KPNA2 is a nuclear export protein that contributes to aberrant localisation of key proteins and poor prognosis of breast cancer. Br J Cancer.

[34] Lin JR, Hu J (2013). SeqNLS: nuclear localization signal prediction based on frequent pattern mining and linear motif scoring. PLoS One.

[35] Li K, Mo C, Gong D, Chen Y, Huang Z, Li Y (2017). DDX17 nucleocytoplasmic shuttling promotes acquired gefitinib resistance in non-small cell lung cancer cells via activation of β-catenin. Cancer Lett.

[36] McLaughlin SK, Olsen SN, Dake B, De Raedt T, Lim E, Bronson RT (2013). The RasGAP gene, RASAL2, is a tumor and metastasis suppressor. Cancer Cell.

[37] Shen J, Wang Y, Hung MC (2013). RASAL2: wrestling in the combat of Ras activation. Cancer Cell.

[38] Olsen SN, Wronski A, Castano Z, Dake B, Malone C, De Raedt T (2017). Loss of RasGAP tumor suppressors underlies the aggressive nature of luminal B breast cancers. Cancer Discov.

[39] Hui K, Gao Y, Huang J, Xu S, Wang B, Zeng J (2017). RASAL2, a RAS GTPase-activating protein, inhibits stemness and epithelial-mesenchymal transition via MAPK/SOX2 pathway in bladder cancer. Cell Death Dis.

[40] Friedrich B, Quensel C, Sommer T, Hartmann E, Kohler M (2006). Nuclear localization signal and protein context both mediate importin alpha specificity of nuclear import substrates. Mol Cell Biol.

[41] Duan J, Tang Z, Mu H, Zhang G (2017). [retracted] nuclear import of prototype foamy virus transactivator bel1 is mediated by KPNA1, KPNA6 and KPNA7. Int J Mol Med.

[42] Ishizawa J, Kojima K, Hail NJ, Tabe Y, Andreeff M (2015). Expression, function, and targeting of the nuclear exporter chromosome region maintenance 1 (CRM1) protein. Pharmacol Ther.

[43] Mahipal A, Malafa M (2016). Importins and exportins as therapeutic targets in cancer. Pharmacol Ther.

[44] Mutka SC, Yang WQ, Dong SD, Ward SL, Craig DA, Timmermans PB (2009). Identification of nuclear export inhibitors with potent anticancer activity in vivo. Cancer Res.

[45] Tai YT, Landesman Y, Acharya C, Calle Y, Zhong MY, Cea M (2014). CRM1 inhibition induces tumor cell cytotoxicity and impairs osteoclastogenesis in multiple myeloma: molecular mechanisms and therapeutic implications. Leukemia.

[46] Hing ZA, Fung HY, Ranganathan P, Mitchell S, El-Gamal D, Woyach JA (2016). Next-generation XPO1 inhibitor shows improved efficacy and in vivo tolerability in hematological malignancies. Leukemia.

[47] Walker CJ, Oaks JJ, Santhanam R, Neviani P, Harb JG, Ferenchak G (2013). Preclinical and clinical efficacy of XPO1/CRM1 inhibition by the karyopherin inhibitor KPT-330 in Ph+ leukemias. Blood.

[48] van der Watt PJ, Chi A, Stelma T, Stowell C, Strydom E, Carden S (2016). Targeting the nuclear import receptor Kpnbeta1 as an anticancer therapeutic. Mol Cancer Ther.

