# Supplementary figures and images for: IPO5 promotes the proliferation and tumourigenicity of colorectal cancer cells by mediating RASAL2 nuclear transportation
Source: J Exp Clin Cancer Res. 2019 Jul 9;38:296. doi: 10.1186/s13046-019-1290-0 (PMC6617704; doi:10.1186/s13046-019-1290-0)

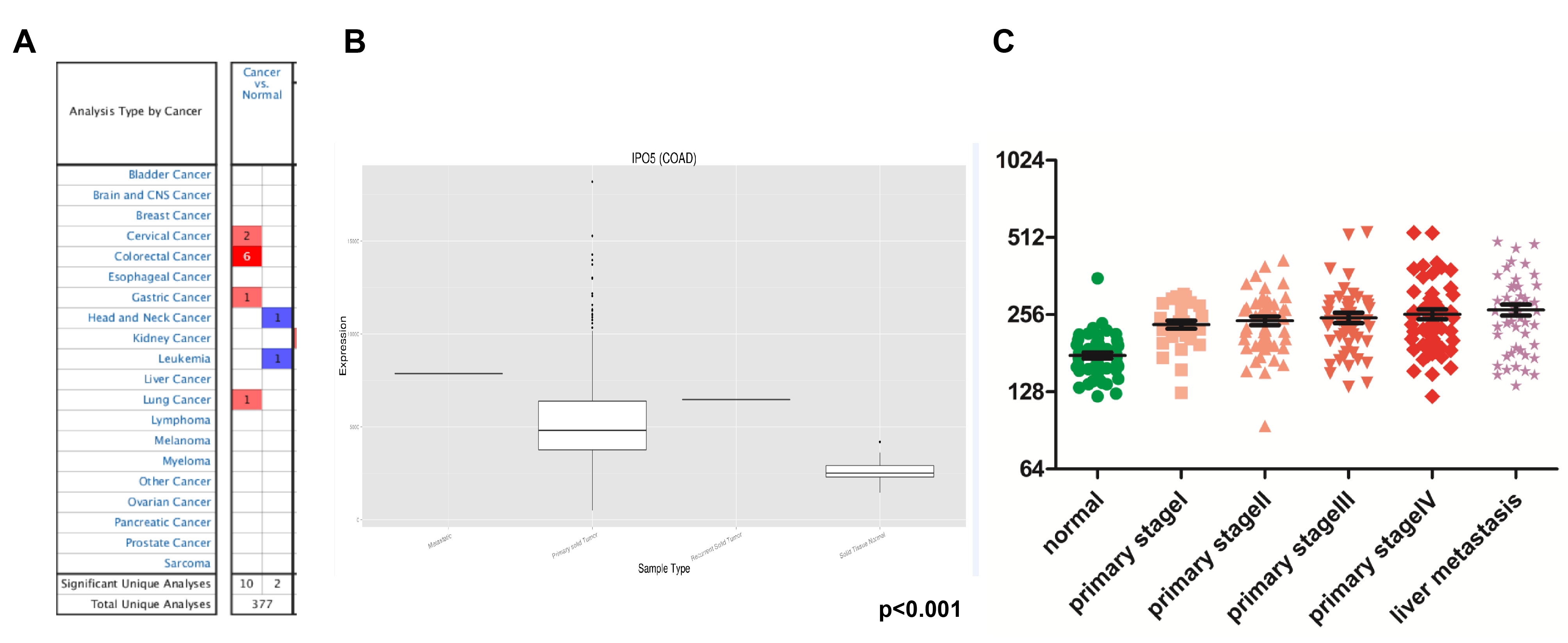

Supplement: Supplementary file 1 — Figure S1. The expression of IPO5, data from public database. (A) Analysis of IPO5 expression in different types of malignancies. (B) Analysis of IPO5 expression in TCGA CRC large sample genomic database. (C) Analysis of IPO5 expression using the CRC gene expression profile data GSE41258. (JPG 849 kb) [file 13046_2019_1290_MOESM1_ESM.jpg]

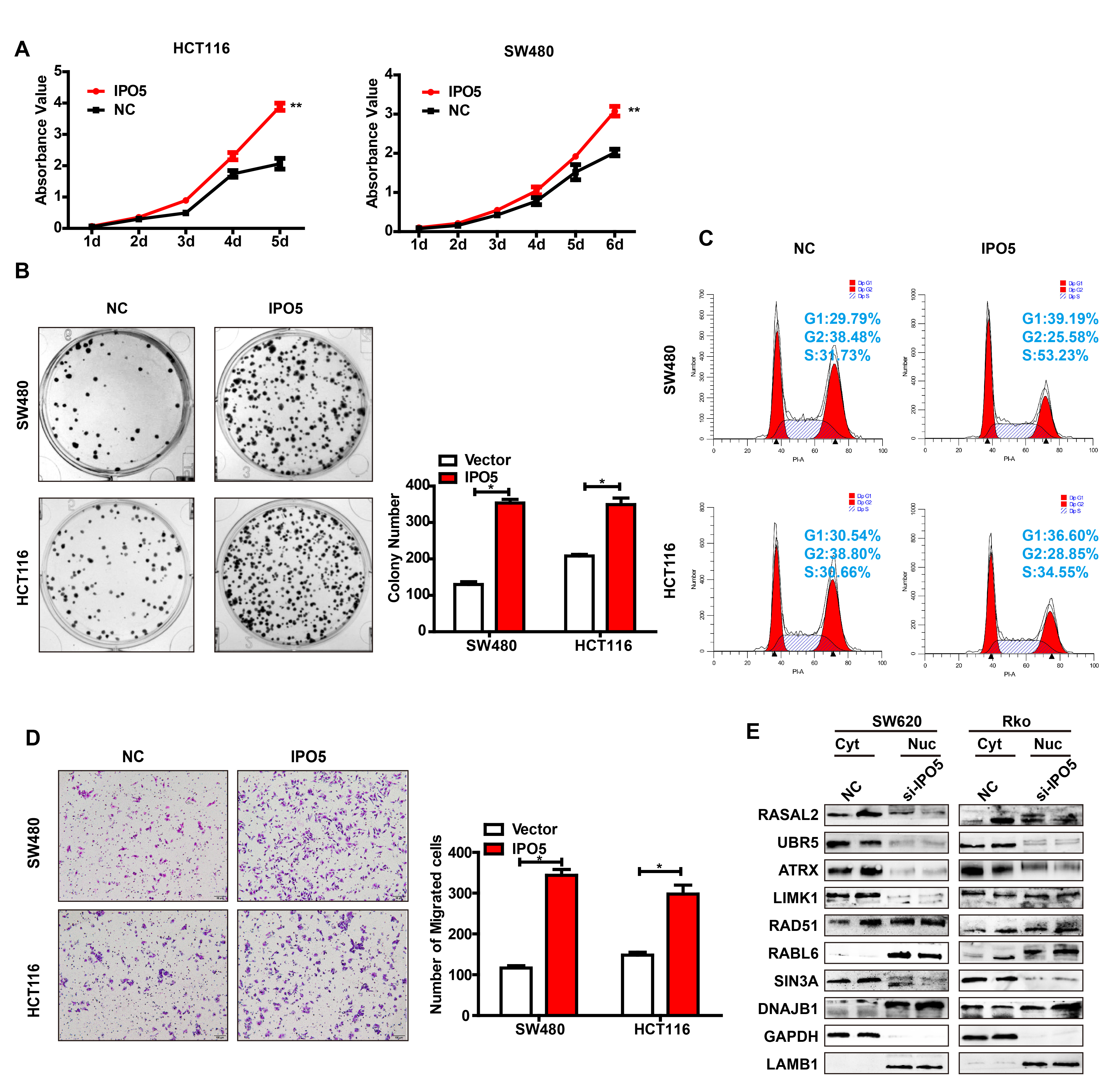

Supplement: Supplementary file 2 — Figure S2. Effects of IPO5 over-expression on CRC cell proliferation and migration in vitro (A and B) Up-regulation of IPO5 increased cell proliferation (P < 0.01) and clonogenicity (P < 0.01) as compared to controls . (C) The effect of IPO5 on the cell cycle distribution was detected by flow cytometry in CRC cells. (D) The impact of ectopic expression of IPO5 on cell migration was validated by transwell assay(P < 0.01). (E) Screen out IPO5 transporting cargos using subcellular fractionation methods followed by immunoblotting. (JPG 5002 kb) [file 13046_2019_1290_MOESM2_ESM.jpg]
