# Supplementary material for: IPO5 promotes the proliferation and tumourigenicity of colorectal cancer cells by mediating RASAL2 nuclear transportation
Source: J Exp Clin Cancer Res. 2019 Jul 9;38:296. doi: 10.1186/s13046-019-1290-0 (PMC6617704; doi:10.1186/s13046-019-1290-0)
Supplement: Supplementary file 3 — Table S1. List of IPO5 binding candidates identified by mass spectrometry. (DOCX 55 kb) [file 13046_2019_1290_MOESM3_ESM.docx]

**Table S1. List of IPO5 binding candidates identified by mass spectrometry.**

| **Gene** | **Accessin** |  | **Description** |  |
| --- | --- | --- | --- | --- |
| TUBA1A | Q71U36 |  | tubulin alpha-1A chain |  |
| ESYT1 | Q9BSJ8 |  | Extended synaptotagmin-1 |  |
| HSPA1L | P34931 |  | Heat shock 70 kDa protein 1-like |  |
| SMPD4 | Q9NXE4-2 |  | Isoform 2 of Sphingomyelin phosphodiesterase 4 |  |
| KRT16 | P08779 |  | Keratin, type I cytoskeletal 16 |  |
| ACTR2 | P61160-1 |  | Actin-related protein 2 |  |
| DEF6 | Q9H4E7 |  | differentially expressed in FDCP 6 homolog |  |
| SEC61A2 | Q9H9S3 |  | Protein transport protein Sec61 subunit alpha isoform 2 |  |
| HLA-F | P30511-3 |  | Isoform 3 of HLA class I histocompatibility antigen, alpha chain F |  |
| PABPC4 | Q13310-3 |  | Isoform 3 of Polyadenylate-binding protein 4 |  |
| RPS3 | P23396-2 |  | Isoform 2 of 40S ribosomal protein S3 |  |
| HNRNPAB | Q99729-2 |  | Isoform 2 of Heterogeneous nuclear ribonucleoprotein A/B |  |
| PDK1 | Q15118 |  | [Pyruvate dehydrogenase (Acetyl-transferring)] kinase isozyme 1, mitochondrial |  |
| GNAI1 | P63096-1 |  | Guanine nucleotide-binding protein G(i) subunit alpha-1 |  |
| HLA-E | P13747 |  | HLA class I histocompatibility antigen, alpha chain E |  |
| PUM2 | Q8TB72-1 |  | pumilio homolog 2 |  |
| CYFIP2 | Q96F07 |  | Cytoplasmic FMR1-interacting protein 2 |  |
| INPP1 | P49441 |  | Inositol polyphosphate 1-phosphatase |  |
| M6PR | P20645 |  | Cation-dependent mannose-6-phosphate receptor |  |
| CCNB2 | O95067 |  | G2/mitotic-specific cyclin-B2 |  |
| MRPL44 | Q9H9J2 |  | 39S ribosomal protein L44, mitochondrial |  |
| NGEF | Q8N5V2-1 |  | Ephexin-1 |  |
| CDK3 | Q00526 |  | Cyclin-dependent kinase 3 |  |
| CASKIN2 | Q8WXE0-1 |  | caskin-2 |  |
| HSPA4 | P34932 |  | Heat shock 70 kDa protein 4 |  |
| ATP1B3 | P54709 |  | sodium/potassium-transporting ATPase subunit beta-3 |  |
| TGFBRAP1 | Q8WUH2 |  | Transforming growth factor-beta receptor-associated protein 1 |  |
| UGCG | Q16739 |  | ceramide glucosyltransferase |  |
| PLXNB2 | O15031 |  | Plexin-B2 |  |
| NEK7 | Q8TDX7-1 |  | Serine/threonine-protein kinase Nek7 |  |
| KDELR2 | P33947-1 |  | ER lumen protein-retaining receptor 2 |  |
| HEATR5A | Q86XA9 |  | HEAT repeat-containing protein 5A |  |
| MTMR3 | Q13615 |  | myotubularin-related protein 3 |  |
| CDC45 | O75419-3 |  | Isoform 3 of Cell division control protein 45 homolog |  |
| TLK1 | Q9UKI8-2 |  | Isoform 2 of Serine/threonine-protein kinase tousled-like 1 |  |
| GCDH | Q92947-1 |  | Glutaryl-CoA dehydrogenase, mitochondrial |  |
| CANX | P27824-2 |  | Isoform 2 of Calnexin |  |
| ABHD13 | Q7L211 |  | Protein Abhd13 |  |
| WDR75 | Q8IWA0 |  | WD repeat-containing protein 75 |  |
| TOR2A | Q5JU69-1 |  | Torsin-2A |  |
| NT5C3B | Q969T7 |  | 7-methylguanosine phosphate-specific 5'-nucleotidase |  |
| GPAT3 | Q53EU6 |  | Glycerol-3-phosphate acyltransferase 3 |  |
| GTF3C2 | Q8WUA4-1 |  | General transcription factor 3C polypeptide 2 |  |
| ALDH16A1 | Q8IZ83 |  | Aldehyde dehydrogenase family 16 member A1 |  |
| SBF2 | Q86WG5 |  | Myotubularin-related protein 13 |  |
| TOP2B | Q02880 |  | DNA topoisomerase 2-beta |  |
| ARRB2 | P32121-4 |  | Isoform 4 of Beta-arrestin-2 |  |
| FERMT3 | Q86UX7 |  | Fermitin family homolog 3 |  |
| MUL1 | Q969V5 |  | Mitochondrial ubiquitin ligase activator of NFKB 1 |  |
| ZGPAT | Q8N5A5-1 |  | Zinc finger CCCH-type with G patch domain-containing protein |  |
| IFIT3 | O14879 |  | Interferon-induced protein with tetratricopeptide repeats 3 |  |
| APH1A | Q96BI3-1 |  | gamma-secretase subunit APH-1A |  |
| CPNE5 | Q9HCH3 |  | Copine-5 |  |
| RGL3 | Q3MIN7-2 |  | Isoform 2 of Ral guanine nucleotide dissociation stimulator-like 3 |  |
| PGM2 | Q96G03 |  | Phosphoglucomutase-2 |  |
| DDX27 | Q96GQ7 |  | Probable ATP-dependent RNA helicase DDX27 |  |
| HPS5 | Q9UPZ3 |  | Hermansky-Pudlak syndrome 5 protein |  |
| PCDHB8 | Q9UN66 |  | Protocadherin beta-8 |  |
| KIF1C | O43896 |  | Kinesin-like protein KIF1C |  |
| PRKAG2 | Q9UGJ0 |  | 5'-AMP-activated protein kinase subunit gamma-2 |  |
| MRS2 | Q9HD23 |  | magnesium transporter MRS2 homolog, mitochondrial |  |
| CDK7 | P50613 |  | Cyclin-dependent kinase 7 |  |
| ARIH1 | Q9Y4X5 |  | E3 ubiquitin-protein ligase ARIH1 |  |
| RIOK1 | Q9BRS2 |  | Serine/threonine-protein kinase RIO1 |  |
| BABAM2 | Q9NXR7-1 |  | Isoform 1 of BRCA1-A complex subunit BRE |  |
| SLC27A1 | Q6PCB7 |  | long-chain fatty acid transport protein 1 |  |
| RAD51 | Q06609-4 |  | Isoform 4 of DNA repair protein RAD51 homolog 1 |  |
| SRSF6 | Q13247 |  | Serine/arginine-rich splicing factor 6 |  |
| MTMR4 | Q9NYA4 |  | Myotubularin-related protein 4 |  |
| ZZEF1 | O43149 |  | Zinc finger ZZ-type and EF-hand domain-containing protein 1 |  |
| RDH13 | Q8NBN7 |  | Retinol dehydrogenase 13 |  |
| UBR5 | O95071 |  | E3 ubiquitin-protein ligase UBR5 |  |
| ARMC8 | Q8IUR7-1 |  | Armadillo repeat-containing protein 8 |  |
| TXNL1 | O43396 |  | Thioredoxin-like protein 1 |  |
| DDX11 | Q96FC9-1 |  | Probable ATP-dependent DNA helicase DDX11 |  |
| TRA2A | Q13595-1 |  | Transformer-2 protein homolog alpha |  |
| SUGP2 | Q8IX01-1 |  | SURP and G-patch domain-containing protein 2 |  |
| SARAF | Q96BY9 |  | Store-operated calcium entry-associated regulatory factor |  |
| CLPTM1 | O96005-1 |  | Cleft lip and palate transmembrane protein 1 |  |
| UBE2Q1 | Q7Z7E8-1 |  | Ubiquitin-conjugating enzyme E2 Q1 |  |
| C12ORF29 | Q8N999-1 |  | Uncharacterized protein C12orf29 |  |
| LIMK1 | P53667-1 |  | LIM domain kinase 1 |  |
| SLC35A1 | P78382 |  | CMP-sialic acid transporter |  |
| SEH1L | Q96EE3-1 |  | Isoform B of Nucleoporin SEH1 |  |
| SLC39A14 | Q15043 |  | Zinc transporter ZIP14 |  |
| FBXL4 | Q9UKA2 |  | F-box/LRR-repeat protein 4 |  |
| BCL2L2 | Q92843-2 |  | Isoform 3 of Bcl-2-like protein 2 |  |
| N4BP3 | O15049 |  | NEDD4-binding protein 3 |  |
| MICAL1 | Q8TDZ2-4 |  | Isoform 4 of Protein-methionine sulfoxide oxidase MICAL1 |  |
| AKAP13 | Q12802-2 |  | Isoform 2 of A-kinase anchor protein 13 |  |
| KDM2A | Q9Y2K7 |  | Lysine-specific demethylase 2A |  |
| OSGEPL1 | Q9H4B0 |  | Probable tRNA N6-adenosine threonylcarbamoyltransferase, mitochondrial |  |
| GNAQ | P50148 |  | Guanine nucleotide-binding protein G(Q) subunit alpha |  |
| TRMT6 | Q9UJA5-1 |  | tRNA (adenine(58)-N(1))-methyltransferase non-catalytic subunit TRM6 |  |
| RANBP10 | Q6VN20 |  | Ran-binding protein 10 |  |
| EML3 | Q32P44 |  | Echinoderm microtubule-associated protein-like 3 |  |
| RRP7A | Q9Y3A4 |  | Ribosomal RNA-processing protein 7 homolog A |  |
| SLC7A2 | P52569-3 |  | Isoform 3 of Cationic amino acid transporter 2 |  |
| SIN3A | Q96ST3 |  | Paired amphipathic helix protein Sin3a |  |
| CUL3 | Q13618 |  | Cullin-3 |  |
| DNAJB1 | P25685 |  | dnaJ homolog subfamily B member 1 |  |
| PRKAB1 | Q9Y478 |  | 5'-AMP-activated protein kinase subunit beta-1 |  |
| SMG9 | Q9H0W8 |  | protein SMG9 |  |
| SF3B4 | Q15427 |  | Splicing factor 3b subunit 4 |  |
| MRPL38 | Q96DV4 |  | 39S ribosomal protein L38, mitochondrial |  |
| PRMT7 | Q9NVM4 |  | Protein arginine N-methyltransferase 7 |  |
| IFI16 | Q16666-1 |  | gamma-interferon-inducible protein 16 |  |
| SNX13 | Q9Y5W8 |  | Sorting nexin-13 |  |
| POLR2H | P52434-4 |  | Isoform 4 of DNA-directed RNA polymerases I, II, and III subunit RPABC3 |  |
| ATAD1 | Q8NBU5 |  | ATPase family aaa domain-containing protein 1 |  |
| COPS4 | Q9BT78 |  | COP9 signalosome complex subunit 4 |  |
| TMEM106B | Q9NUM4 |  | Transmembrane protein 106B |  |
| TRIM33 | Q9UPN9-1 |  | E3 ubiquitin-protein ligase TRIM33 |  |
| GGCX | P38435-1 |  | vitamin K-dependent gamma-carboxylase |  |
| IVNS1ABP | Q9Y6Y0 |  | influenza virus NS1A-binding protein |  |
| JUP | P14923 |  | Junction plakoglobin |  |
| MON2 | Q7Z3U7-1 |  | Protein MON2 homolog |  |
| VKORC1L1 | Q8N0U8 |  | Vitamin K epoxide reductase complex subunit 1-like protein 1 |  |
| SSFA2 | P28290 |  | Sperm-specific antigen 2 |  |
| ZMYM3 | Q14202 |  | Zinc finger MYM-type protein 3 |  |
| UTP6 | Q9NYH9 |  | U3 small nucleolar RNA-associated protein 6 homolog |  |
| UFD1 | Q92890-1 |  | Isoform Long of Ubiquitin fusion degradation protein 1 homolog |  |
| CNNM3 | Q8NE01 |  | Metal transporter CNNM3 |  |
| CORO2B | Q9UQ03-1 |  | Coronin-2B |  |
| TSC1 | Q92574-1 |  | Hamartin |  |
| STAU2 | Q9NUL3 |  | Double-stranded RNA-binding protein Staufen homolog 2 |  |
| CLN8 | Q9UBY8 |  | protein CLN8 |  |
| TOP2A | P11388-4 |  | Isoform 4 of DNA topoisomerase 2-alpha |  |
| ETFDH | Q16134 |  | Electron transfer flavoprotein-ubiquinone oxidoreductase, mitochondrial |  |
| AHCY | P23526-1 |  | Adenosylhomocysteinase |  |
| L3MBTL3 | Q96JM7 |  | Lethal(3)malignant brain tumor-like protein 3 |  |
| USP18 | Q9UMW8 |  | Ubl carboxyl-terminal hydrolase 18 |  |
| TTPAL | Q9BTX7 |  | Alpha-tocopherol transfer protein-like |  |
| HNRNPUL2 | Q1KMD3 |  | heterogeneous nuclear ribonucleoprotein U-like protein 2 |  |
| NPRL2 | Q8WTW4 |  | Nitrogen permease regulator 2-like protein |  |
| LDAH | Q9H6V9-2 |  | Isoform 2 of Lipid droplet-associated hydrolase |  |
| ARHGEF40 | Q8TER5 |  | Rho guanine nucleotide exchange factor 40 |  |
| ARHGEF10L | Q9HCE6 |  | Rho guanine nucleotide exchange factor 10-like protein |  |
| PTPRS | Q13332 |  | Receptor-type tyrosine-protein phosphatase S |  |
| MYO19 | Q96H55-1 |  | unconventional myosin-XIX |  |
| NDUFAF5 | Q5TEU4 |  | NADH dehydrogenase [ubiquinone] 1 alpha subcomplex assembly factor 5 |  |
| ATRX | P46100 |  | transcriptional regulator ATRX |  |
| BRWD1 | Q9NSI6-1 |  | bromodomain and WD repeat-containing protein 1 |  |
| PLEKHH3 | Q7Z736 |  | Pleckstrin homology domain-containing family H member 3 |  |
| SNX25 | Q9H3E2 |  | Sorting nexin-25 |  |
| AHSG | P02765 |  | Alpha-2-HS-glycoprotein |  |
| WNK1 | Q9H4A3-7 |  | Isoform 6 of Serine/threonine-protein kinase WNK1 |  |
| NLGN1 | Q8N2Q7-1 |  | Neuroligin-1 |  |
| B3GNT2 | Q9NY97-1 |  | N-acetyllactosaminide beta-1,3-N-acetylglucosaminyltransferase 2 |  |
| SAFB | Q15424-3 |  | Isoform 3 of Scaffold attachment factor B1 |  |
| USP11 | P51784 |  | Ubiquitin carboxyl-terminal hydrolase 11 |  |
| COG8 | Q96MW5 |  | Conserved oligomeric Golgi complex subunit 8 |  |
| FECH | P22830-2 |  | Isoform 2 of Ferrochelatase, mitochondrial |  |
| TCTN3 | Q6NUS6 |  | tectonic-3 |  |
| UBP1 | Q9NZI7 |  | Upstream-binding protein 1 |  |
| LRIT3 | Q3SXY7-1 |  | Leucine-rich repeat, immunoglobulin-like domain and transmembrane domain-containing protein 3 |  |
| ENGASE | Q8NFI3-1 |  | Cytosolic endo-beta-N-acetylglucosaminidase |  |
| PJA2 | O43164 |  | E3 ubiquitin-protein ligase Praja-2 |  |
| LMBRD1 | Q9NUN5-1 |  | Probable lysosomal cobalamin transporter |  |
| PC | P11498 |  | pyruvate carboxylase, mitochondrial |  |
| ESF1 | Q9H501 |  | ESF1 homolog |  |
| LAMP2 | P13473-3 |  | Isoform LAMP-2C of Lysosome-associated membrane glycoprotein 2 |  |
| MARK3 | P27448-5 |  | MAP/microtubule affinity-regulating kinase 3 |  |
| PHLDB3 | Q6NSJ2-1 |  | Pleckstrin homology-like domain family B member 3 |  |
| HSPA13 | P48723 |  | Heat shock 70 kDa protein 13 |  |
| SON | P18583-5 |  | Isoform D of Protein SON |  |
| CRYBG3 | Q68DQ2 |  | very large A-kinase anchor protein |  |
| SLC39A10 | Q9ULF5 |  | Zinc transporter ZIP10 |  |
| CCDC88A | Q3V6T2-1 |  | Girdin |  |
| ORC2 | Q13416 |  | Origin recognition complex subunit 2 |  |
| TCEA1 | P23193 |  | Transcription elongation factor A protein 1 |  |
| MIB1 | Q86YT6 |  | E3 ubiquitin-protein ligase mib1 |  |
| CUX1 | P39880-3 |  | Isoform 3 of Homeobox protein cut-like 1 |  |
| RDH14 | Q9HBH5 |  | Retinol dehydrogenase 14 |  |
| DOCK4 | Q8N1I0 |  | Dedicator of cytokinesis protein 4 |  |
| CARMIL1 | Q5VZK9 |  | F-actin-uncapping protein LRRC16A |  |
| GLG1 | Q92896-2 |  | Isoform 2 of Golgi apparatus protein 1 |  |
| MKRN2 | Q9H000 |  | Probable E3 ubiquitin-protein ligase makorin-2 |  |
| KTN1 | Q86UP2-1 |  | Kinectin |  |
| PPIE | Q9UNP9-3 |  | Isoform 3 of Peptidyl-prolyl cis-trans isomerase E |  |
| TOE1 | Q96GM8 |  | Target of EGR1 protein 1 |  |
| PRSS1 | P07477 |  | Trypsin-1 |  |
| RIOK2 | Q9BVS4 |  | Serine/threonine-protein kinase rio2 |  |
| BUB1B | O60566-3 |  | Isoform 3 of Mitotic checkpoint serine/threonine-protein kinase BUB1 beta |  |
| ZC3H4 | Q9UPT8 |  | Zinc finger CCCH domain-containing protein 4 |  |
| SLC20A2 | Q08357 |  | sodium-dependent phosphate transporter 2 |  |
| UBA2 | Q9UBT2 |  | SUMO-activating enzyme subunit 2 |  |
| CLSTN3 | Q9BQT9-2 |  | Isoform 2 of Calsyntenin-3 |  |
| PIK3CA | P42336 |  | Phosphatidylinositol 4,5-bisphosphate 3-kinase catalytic subunit alpha isoform |  |
| RABL6 | Q3YEC7-2 |  | Isoform 2 of Rab-like protein 6 |  |
| KEAP1 | Q14145 |  | Kelch-like ECH-associated protein 1 |  |
| ATAD2B | Q9ULI0 |  | ATPase family AAA domain-containing protein 2B |  |
| HMOX2 | P30519 |  | Heme oxygenase 2 |  |
| FAM118B | Q9BPY3 |  | Protein FAM118B |  |
| SPNS1 | Q9H2V7 |  | Protein spinster homolog 1 |  |
| RASAL2 | Q9UJF2-2 |  | Isoform 2 of Ras GTPase-activating protein nGAP |  |
| TTC39B | Q5VTQ0-1 |  | Tetratricopeptide repeat protein 39B |  |
| ZMYM4 | Q5VZL5 |  | Zinc finger MYM-type protein 4 |  |
| CORO1C | Q9ULV4-3 |  | Isoform 3 of Coronin-1C |  |
| PIGW | Q7Z7B1 |  | Phosphatidylinositol-glycan biosynthesis class W protein |  |
| MAEA | Q7L5Y9-1 |  | Macrophage erythroblast attacher |  |
| USP38 | Q8NB14 |  | Ubiquitin carboxyl-terminal hydrolase 38 |  |
| GCC1 | Q96CN9 |  | GRIP and coiled-coil domain-containing protein 1 |  |
| OSGEP | Q9NPF4 |  | Probable tRNA N6-adenosine threonylcarbamoyltransferase |  |
| NAAA | Q02083 |  | N-acylethanolamine-hydrolyzing acid amidase |  |
| NDRG1 | Q92597 |  | Protein NDRG1 |  |
| CEP170 | Q5SW79-1 |  | Centrosomal protein of 170 kDa |  |
| TCIRG1 | Q13488 |  | V-type proton ATPase 116 kDa subunit a isoform 3 |  |
| SLC48A1 | Q6P1K1 |  | Heme transporter HRG1 |  |
| DOCK11 | Q5JSL3 |  | dedicator of cytokinesis protein 11 |  |
| SLC9A7 | Q96T83 |  | Sodium/hydrogen exchanger 7 |  |
| ST7 | Q9NRC1-1 |  | Suppressor of tumorigenicity 7 protein |  |
| STK11IP | Q8N1F8 |  | Serine/threonine-protein kinase 11-interacting protein |  |
| UHRF1BP1 | Q6BDS2 |  | UHRF1-binding protein 1 |  |
| TBKBP1 | A7MCY6-1 |  | TANK-binding kinase 1-binding protein 1 |  |
| VPS11 | Q9H270 |  | Vacuolar protein sorting-associated protein 11 homolog |  |
| THOC1 | Q96FV9-1 |  | THO complex subunit 1 |  |
| LMAN2 | Q12907 |  | Vesicular integral-membrane protein VIP36 |  |
| PITPNA | Q00169 |  | Phosphatidylinositol transfer protein alpha isoform |  |
| CDK5RAP2 | Q96SN8 |  | CDK5 regulatory subunit-associated protein 2 |  |
| FAM83G | A6ND36-1 |  | Protein FAM83G |  |
| RAE1 | P78406 |  | mRNA export factor |  |
| STIP1 | P31948-2 |  | Isoform 2 of Stress-induced-phosphoprotein 1 |  |
| UBE3B | Q7Z3V4-1 |  | Ubiquitin-protein ligase E3B |  |
| EP400 | Q96L91 |  | E1A-binding protein p400 |  |
| CUL7 | Q14999-2 |  | Isoform 2 of Cullin-7 |  |
| AMDHD2 | Q9Y303-3 |  | Isoform 3 of N-acetylglucosamine-6-phosphate deacetylase |  |
| TMEM87B | Q96K49-1 |  | Transmembrane protein 87B |  |
| AKAP9 | Q99996-6 |  | Isoform 6 of A-kinase anchor protein 9 |  |
| PDK4 | Q16654 |  | [Pyruvate dehydrogenase (acetyl-transferring)] kinase isozyme 4, mitochondrial |  |
| GET4 | Q7L5D6 |  | Golgi to ER traffic protein 4 homolog |  |
| PCNT | O95613 |  | Pericentrin |  |
| PRTG | Q2VWP7 |  | protogenin |  |
| ALS2 | Q96Q42-1 |  | Alsin |  |
| ANKHD1 | Q8IWZ3-6 |  | Isoform 6 of Ankyrin repeat and KH domain-containing protein 1 |  |
| DHX35 | Q9H5Z1 |  | Probable ATP-dependent RNA helicase DHX35 |  |
| HADH | Q16836-2 |  | Isoform 2 of Hydroxyacyl-coenzyme A dehydrogenase, mitochondrial |  |
| LCMT2 | O60294 |  | tRNA wybutosine-synthesizing protein 4 |  |
| RNF216 | Q9NWF9-1 |  | Isoform 2 of E3 ubiquitin-protein ligase RNF216 |  |
| NBEAL1 | Q6ZS30-2 |  | Neurobeachin-like protein 1 |  |
| ABCC10 | Q5T3U5 |  | Multidrug resistance-associated protein 7 |  |
| CCDC175 | P0C221 |  | Coiled-coil domain-containing protein 175 |  |
| PRMT9 | Q6P2P2 |  | putative protein arginine N-methyltransferase 9 |  |
| PIPSL | A2A3N6 |  | Putative PIP5K1A and PSMD4-like protein |  |
| SYNE1 | Q8NF91 |  | Nesprin-1 |  |
| DSP | P15924-1 |  | Desmoplakin |  |
| TM9SF4 | Q92544 |  | Transmembrane 9 superfamily member 4 |  |
| KDM5A | P29375-1 |  | Lysine-specific demethylase 5A |  |
| PDE4DIP | Q5VU43-4 |  | Isoform 4 of Myomegalin |  |
| SEMA3A | Q14563 |  | Semaphorin-3A |  |
| GLB1 | P16278 |  | Beta-galactosidase |  |
| KLHL22 | Q53GT1 |  | Kelch-like protein 22 |  |
| PKD1L3 | Q7Z443 |  | Polycystic kidney disease protein 1-like 3 |  |
| OGG1 | O15527-4 |  | Isoform 2A of N-glycosylase/DNA lyase |  |
| TMEM131L | A2VDJ0-5 |  | Isoform 4 of Transmembrane protein 131-like |  |
| FBXL19 | Q6PCT2-1 |  | F-box/LRR-repeat protein 19 |  |
| TYK2 | P29597 |  | Non-receptor tyrosine-protein kinase TYK2 |  |
| PKD1L1 | Q8TDX9 |  | Polycystic kidney disease protein 1-like 1 |  |
| C17orf82 | Q86X59 |  | Putative uncharacterized protein C17orf82 |  |
| TCERG1 | O14776-1 |  | Transcription elongation regulator 1 |  |
| DNAJB4 | Q9UDY4 |  | DnaJ homolog subfamily B member 4 |  |
| XDH | P47989 |  | Xanthine dehydrogenase/oxidase |  |
| SENP1 | Q9P0U3-1 |  | Sentrin-specific protease 1 |  |
| MGAM2 | Q2M2H8 |  | Probable maltase-glucoamylase 2 |  |
| PLAUR | Q03405-2 |  | Isoform 2 of Urokinase plasminogen activator surface receptor |  |
| OBSL1 | O75147 |  | Obscurin-like protein 1 |  |
| OPHN1 | O60890 |  | Oligophrenin-1 |  |
| PCGF2 | P35227 |  | Polycomb group RING finger protein 2 |  |
| TYW1 | Q9NV66 |  | S-adenosyl-L-methionine-dependent tRNA 4-demethylwyosine synthase |  |
| H2AFJ | Q9BTM1-2 |  | Isoform 2 of Histone H2A.J |  |
| USP36 | Q9P275-2 |  | Isoform 2 of Ubiquitin carboxyl-terminal hydrolase 36 |  |
| NDC80 | O14777 |  | Kinetochore protein NDC80 homolog |  |
| ULK4 | Q96C45 |  | Serine/threonine-protein kinase ULK4 |  |
| TMEM62 | Q0P6H9 |  | Transmembrane protein 62 |  |
| CAMTA2 | O94983-3 |  | Isoform 3 of Calmodulin-binding transcription activator 2 |  |
| CLRN2 | A0PK11 |  | Clarin-2 |  |
| SLC32A1 | Q9H598 |  | vesicular inhibitory amino acid transporter |  |
| ARL6IP1 | Q15041 |  | ADP-ribosylation factor-like protein 6-interacting protein 1 |  |
| UBA1 | P22314 |  | Ubiquitin-like modifier-activating enzyme 1 |  |
| SPPL2A | Q8TCT8 |  | Signal peptide peptidase-like 2A |  |
| GMPR2 | Q9P2T1-2 |  | Isoform 2 of GMP reductase 2 |  |
| RNF24 | Q9Y225-2 |  | Isoform 2 of RING finger protein 24 |  |
| FLG2 | Q5D862 |  | Filaggrin-2 |  |
| SIK3 | Q9Y2K2 |  | Serine/threonine-protein kinase SIK3 |  |
| MLLT6 | P55198 |  | Protein AF-17 |  |
| ATIC | P31939 |  | bifunctional purine biosynthesis protein purH |  |
| CLIC5 | Q9NZA1-1 |  | Chloride intracellular channel protein 5 |  |
| DDX60L | Q5H9U9-1 |  | Probable ATP-dependent RNA helicase DDX60-like |  |
| PKP2 | Q99959 |  | Plakophilin-2 |  |
| MIGA2 | Q7L4E1-1 |  | Mitoguardin-2 |  |
| LRP4 | O75096 |  | Low-density lipoprotein receptor-related protein 4 |  |
| DDX19B | Q9UMR2-1 |  | ATP-dependent RNA helicase DDX19B |  |
| NDUFA13 | Q9P0J0-2 |  | Isoform 2 of NADH dehydrogenase [ubiquinone] 1 alpha subcomplex subunit 13 |  |
| CISD1 | Q9NZ45 |  | CDGSH iron-sulfur domain-containing protein 1 |  |
| NDUFAF4 | Q9P032 |  | NADH dehydrogenase [ubiquinone] 1 alpha subcomplex assembly factor 4 |  |
| RBM45 | Q8IUH3-3 |  | Isoform 3 of RNA-binding protein 45 |  |
| PPP2R2D | Q66LE6 |  | Serine/threonine-protein phosphatase 2A 55 kDa regulatory subunit B delta isoform |  |
| RAB6A | P20340-2 |  | Isoform 2 of Ras-related protein Rab-6A |  |
| ARHGEF1 | Q92888-3 |  | Isoform 3 of Rho guanine nucleotide exchange factor 1 |  |
| CFL2 | Q9Y281-1 |  | Cofilin-2 |  |
| RAB18 | Q9NP72-2 |  | Isoform 2 of Ras-related protein Rab-18 |  |
| IRF3 | Q14653 |  | interferon regulatory factor 3 |  |
| RRAS | P10301 |  | Ras-related protein R-Ras |  |
| RPS17 | P08708 |  | 40S ribosomal protein S17 |  |
| CBWD2 | Q8IUF1 |  | COBW domain-containing protein 2 |  |
| MRPS11 | P82912-1 |  | 28S ribosomal protein S11, mitochondrial |  |
| AP1S1 | P61966 |  | AP-1 complex subunit sigma-1A |  |
| UCHL3 | P15374 |  | Ubiquitin carboxyl-terminal hydrolase isozyme L3 |  |
| RNF40 | O75150 |  | E3 ubiquitin-protein ligase BRE1B |  |
| DBN1 | Q16643-3 |  | Isoform 3 of Drebrin |  |
| SRP19 | P09132-1 |  | Signal recognition particle 19 kDa protein |  |
| DYNLL2 | Q96FJ2 |  | Dynein light chain 2, cytoplasmic |  |
| PPP3CB | P16298-4 |  | Isoform 4 of Serine/threonine-protein phosphatase 2B catalytic subunit beta isoform |  |
| SRSF1 | Q07955-2 |  | Isoform ASF-2 of Serine/arginine-rich splicing factor 1 |  |
| PSMG1 | O95456 |  | Proteasome assembly chaperone 1 |  |
| ANKS1A | Q92625 |  | Ankyrin repeat and SAM domain-containing protein 1A |  |
| PRKRA | O75569-1 |  | Interferon-inducible double-stranded RNA-dependent protein kinase activator A |  |
| PICALM | Q13492-1 |  | Phosphatidylinositol-binding clathrin assembly protein |  |
| TPMT | P51580 |  | Thiopurine S-methyltransferase |  |
| GPS1 | Q13098-7 |  | Isoform 2 of COP9 signalosome complex subunit 1 |  |
| VASP | P50552 |  | Vasodilator-stimulated phosphoprotein |  |
| RAB43 | Q86YS6 |  | Ras-related protein Rab-43 |  |
| FAM98B | Q52LJ0-2 |  | Isoform 2 of Protein FAM98B |  |
| COPS7A | Q9UBW8 |  | COP9 signalosome complex subunit 7a |  |
| TIMM13 | Q9Y5L4 |  | mitochondrial import inner membrane translocase subunit TIM13 |  |
| CDK17 | Q00537 |  | cyclin-dependent kinase 17 |  |
| RNF2 | Q99496 |  | E3 ubiquitin-protein ligase RING2 |  |
| RALA | P11233 |  | Ras-related protein Ral-A |  |
| ITGB1 | P05556-3 |  | Isoform 3 of Integrin beta-1 |  |
| CAMK2D | Q13557-11 |  | Isoform Delta 11 of Calcium/calmodulin-dependent protein kinase type II subunit delta |  |
| MPV17L2 | Q567V2-1 |  | Mpv17-like protein 2 |  |
| HSPA6 | P17066 |  | Heat shock 70 kDa protein 6 |  |
| GANAB | Q14697-2 |  | Isoform 2 of Neutral alpha-glucosidase AB |  |
| MED22 | Q15528-1 |  | Mediator of RNA polymerase II transcription subunit 22 |  |
| RPS24 | P62847-4 |  | Isoform 4 of 40S ribosomal protein S24 |  |
| DTX3L | Q8TDB6-1 |  | E3 ubiquitin-protein ligase DTX3L |  |
| PNKD | Q8N490-2 |  | Isoform 2 of Probable hydrolase PNKD |  |
| NDUFS6 | O75380 |  | NADH dehydrogenase [ubiquinone] iron-sulfur protein 6, mitochondrial |  |
| DDX54 | Q8TDD1-2 |  | Isoform 2 of ATP-dependent RNA helicase DDX54 |  |
| IGKC | P01834 |  | Ig kappa chain C region |  |
| METTL26 | Q96S19 |  | UPF0585 protein C16orf13 |  |
| NSUN5 | Q96P11-2 |  | Isoform 2 of Probable 28S rRNA (cytosine-C(5))-methyltransferase |  |
| MRPL19 | P49406 |  | 39S ribosomal protein L19, mitochondrial |  |
| SURF1 | Q15526 |  | Surfeit locus protein 1 |  |
| PDCD4 | Q53EL6-1 |  | Programmed cell death protein 4 |  |
| ATL1 | Q8WXF7 |  | Atlastin-1 |  |
| LSM12 | Q3MHD2-2 |  | Isoform 2 of Protein LSM12 homolog |  |
| IFIT1B | Q5T764 |  | Interferon-induced protein with tetratricopeptide repeats 1B |  |
| RAB29 | O14966-1 |  | Ras-related protein Rab-7L1 |  |
| CNBP | P62633-6 |  | Isoform 6 of Cellular nucleic acid-binding protein |  |
| BID | P55957-2 |  | Isoform 2 of BH3-interacting domain death agonist |  |
| DCTN5 | Q9BTE1 |  | Dynactin subunit 5 |  |
| PTBP3 | O95758-4 |  | Isoform 4 of Polypyrimidine tract-binding protein 3 |  |
| NR2C2AP | Q86WQ0-2 |  | Isoform 2 of Nuclear receptor 2C2-associated protein |  |
| FRG1 | Q14331 |  | Protein FRG1 |  |
| CDC7 | O00311-1 |  | Cell division cycle 7-related protein kinase |  |
| NDUFAF3 | Q9BU61-1 |  | NADH dehydrogenase [ubiquinone] 1 alpha subcomplex assembly factor 3 |  |
| TACO1 | Q9BSH4 |  | Translational activator of cytochrome c oxidase 1 |  |
| RPL32 | P62910 |  | 60S ribosomal protein L32 |  |
| OXNAD1 | Q96HP4 |  | Oxidoreductase NAD-binding domain-containing protein 1 |  |
| IK | Q13123 |  | Protein Red |  |
| UCK1 | Q9HA47-4 |  | Isoform 4 of Uridine-cytidine kinase 1 |  |
| MYO6 | Q9UM54-6 |  | Isoform 6 of Unconventional myosin-VI |  |
| KRT18 | P05783 |  | Keratin, type I cytoskeletal 18 |  |
| FAM162A | Q96A26 |  | Protein FAM162A |  |
| ATP6AP1 | Q15904 |  | V-type proton ATPase subunit S1 |  |
| SLC25A21 | Q9BQT8 |  | Mitochondrial 2-oxodicarboxylate carrier |  |
| RPRD1B | Q9NQG5 |  | regulation of nuclear pre-mRNA domain-containing protein 1B |  |
| SNRPA | P09012 |  | U1 SMALL NUCLEAR RIBONUCLEOPROTEIN A |  |
| METTL1 | Q9UBP6 |  | tRNA (guanine-N(7)-)-methyltransferase |  |
| MEMO1 | Q9Y316-3 |  | Isoform 3 of Protein MEMO1 |  |
| TAF9 | Q16594 |  | transcription initiation factor TFIID subunit 9 |  |
| CDKN1A | P38936 |  | Cyclin-dependent kinase inhibitor 1 |  |
| RPL35A | P18077 |  | 60S ribosomal protein L35a |  |
| PIAS1 | O75925-2 |  | Isoform 2 of E3 SUMO-protein ligase PIAS1 |  |
| SELENOT | P62341 |  | Selenoprotein T |  |
| NRAS | P01111 |  | GTPase NRas |  |
| C7ORF43 | Q8WVR3 |  | Uncharacterized protein C7orf43 |  |
| MED11 | Q9P086 |  | Mediator of RNA polymerase II transcription subunit 11 |  |
| DTX2 | Q86UW9 |  | Probable E3 ubiquitin-protein ligase DTX2 |  |
| CHD3 | Q12873-3 |  | Isoform 3 of Chromodomain-helicase-DNA-binding protein 3 |  |
| MRPL14 | Q6P1L8 |  | 39S ribosomal protein L14, mitochondrial |  |
| HARS2 | P49590 |  | probable histidine--tRNA ligase, mitochondrial |  |
| GPATCH4 | Q5T3I0-3 |  | Isoform 3 of G patch domain-containing protein 4 |  |
| RAD17 | O75943-1 |  | cell cycle checkpoint protein RAD17 |  |
| NIFK | Q9BYG3 |  | MKI67 FHA domain-interacting nucleolar phosphoprotein |  |
| GNG12 | Q9UBI6 |  | guanine nucleotide-binding protein g(i)/g(s)/g(o) subunit gamma-12 |  |
| TAMM41 | Q96BW9-1 |  | Phosphatidate cytidylyltransferase, mitochondrial |  |
| RAP2B | P61225 |  | ras-related protein Rap-2b |  |
| MTPN | P58546 |  | Myotrophin |  |
| COPZ1 | P61923 |  | Coatomer subunit zeta-1 |  |
| SNU13 | P55769 |  | NHP2-like protein 1 |  |
| COL4A3BP | Q9Y5P4-3 |  | Isoform 3 of Collagen type IV alpha-3-binding protein |  |
| RPS28 | P62857 |  | 40S ribosomal protein S28 |  |
| HGS | O14964 |  | Hepatocyte growth factor-regulated tyrosine kinase substrate |  |
| RSBN1L | Q6PCB5-1 |  | Round spermatid basic protein 1-like protein |  |
| RNASEH1 | O60930 |  | Ribonuclease H1 |  |
| EXOSC6 | Q5RKV6 |  | Exosome complex component MTR3 |  |
| SSBP1 | Q04837 |  | Single-stranded DNA-binding protein, mitochondrial |  |
| GOLGA7 | Q7Z5G4-1 |  | Golgin subfamily A member 7 |  |
| ABRAXAS2 | Q15018 |  | BRISC complex subunit Abro1 |  |
| WDR47 | O94967-4 |  | Isoform 4 of WD repeat-containing protein 47 |  |
| COMMD7 | Q86VX2-1 |  | COMM domain-containing protein 7 |  |
| NAA40 | Q86UY6 |  | N-alpha-acetyltransferase 40 |  |
| HMCES | Q96FZ2 |  | embryonic stem cell-specific 5-hydroxymethylcytosine-binding protein |  |
| MAGOHB | Q96A72 |  | protein mago nashi homolog 2 |  |
| QSOX1 | O00391 |  | Sulfhydryl oxidase 1 |  |
| CAT | P04040 |  | catalase |  |
| NDEL1 | Q9GZM8-2 |  | Isoform 2 of Nuclear distribution protein nudE-like 1 |  |
| CPTP | Q5TA50 |  | Ceramide-1-phosphate transfer protein |  |
| PPIL3 | Q9H2H8-2 |  | Isoform 2 of Peptidyl-prolyl cis-trans isomerase-like 3 |  |
| AKAP17A | Q02040-1 |  | A-kinase anchor protein 17A |  |
| LPIN3 | Q9BQK8-2 |  | Isoform 2 of Phosphatidate phosphatase LPIN3 |  |
| GLRX5 | Q86SX6 |  | Glutaredoxin-related protein 5, mitochondrial |  |
| FARP1 | Q9Y4F1-2 |  | Isoform 2 of FERM, RhoGEF and pleckstrin domain-containing protein 1 |  |
| EMG1 | Q92979 |  | Ribosomal RNA small subunit methyltransferase Nep1 |  |
| NDUFA10 | O95299-2 |  | Isoform 2 of NADH dehydrogenase [ubiquinone] 1 alpha subcomplex subunit 10, mitochondrial |  |
| ROGDI | Q9GZN7 |  | Protein rogdi homolog |  |
| CSTB | P04080 |  | Cystatin-B |  |
| PFDN2 | Q9UHV9 |  | Prefoldin subunit 2 |  |
| POLR2J | P52435 |  | DNA-directed RNA polymerase II subunit RPB11-a |  |
| GID8 | Q9NWU2 |  | Glucose-induced degradation protein 8 homolog |  |
| TBPL1 | P62380 |  | TATA box-binding protein-like protein 1 |  |
| UBXN6 | Q9BZV1 |  | UBX domain-containing protein 6 |  |
| ANAPC10 | Q9UM13 |  | Anaphase-promoting complex subunit 10 |  |
| NDUFAF6 | Q330K2-1 |  | NADH dehydrogenase (ubiquinone) complex I, assembly factor 6 |  |
| TMEM205 | Q6UW68 |  | Transmembrane protein 205 |  |
| ELP6 | Q0PNE2 |  | Elongator complex protein 6 |  |
| COMMD5 | Q9GZQ3 |  | COMM domain-containing protein 5 |  |
| C15ORF41 | Q9Y2V0-1 |  | Uncharacterized protein C15orf41 |  |
| PIP5K1C | O60331-3 |  | Isoform 3 of Phosphatidylinositol 4-phosphate 5-kinase type-1 gamma |  |
| KIAA1109 | Q2LD37-1 |  | Uncharacterized protein KIAA1109 |  |
| PSMB10 | P40306 |  | Proteasome subunit beta type-10 |  |
| ZBTB9 | Q96C00 |  | zinc finger and BTB domain-containing protein 9 |  |
| TNRC6B | Q9UPQ9 |  | Trinucleotide repeat-containing gene 6B protein |  |
| IGHG1 | P01857 |  | Ig gamma-1 chain C region |  |
| POP4 | O95707 |  | Ribonuclease P protein subunit p29 |  |
| SIX4 | Q9UIU6 |  | Homeobox protein SIX4 |  |
| CTDNEP1 | O95476 |  | CTD nuclear envelope phosphatase 1 |  |
| ERP29 | P30040-1 |  | Endoplasmic reticulum resident protein 29 |  |
| COX20 | Q5RI15-2 |  | Isoform 2 of Cytochrome c oxidase protein 20 homolog |  |
| PPTC7 | Q8NI37 |  | protein phosphatase ptc7 homolog |  |
| MBD3 | O95983-1 |  | methyl-CpG-binding domain protein 3 |  |
| SLC12A6 | Q9UHW9 |  | Solute carrier family 12 member 6 |  |
| UBAC2 | Q8NBM4-1 |  | Ubiquitin-associated domain-containing protein 2 |  |
| RBMX2 | Q9Y388 |  | RNA-binding motif protein, X-linked 2 |  |
| ANKMY2 | Q8IV38 |  | Ankyrin repeat and MYND domain-containing protein 2 |  |
| MRPL30 | Q8TCC3-2 |  | Isoform 2 of 39S ribosomal protein L30, mitochondrial |  |
| TMEM41A | Q96HV5 |  | Transmembrane protein 41A |  |
| RPS15 | P62841 |  | 40S ribosomal protein S15 |  |
| COMMD2 | Q86X83 |  | COMM domain-containing protein 2 |  |
| PPDPF | Q9H3Y8 |  | Pancreatic progenitor cell differentiation and proliferation factor |  |
| ATXN2L | Q8WWM7-3 |  | Isoform 3 of Ataxin-2-like protein |  |
| COQ10B | Q9H8M1 |  | Coenzyme Q-binding protein COQ10 homolog B, mitochondrial |  |
| CASP3 | P42574 |  | Caspase-3 |  |
| MTERF4 | Q7Z6M4 |  | Transcription termination factor 4, mitochondrial |  |
| DIRAS2 | Q96HU8 |  | GTP-binding protein Di-Ras2 |  |
| NUP54 | Q7Z3B4 |  | Nucleoporin p54 |  |
| NUDT1 | P36639 |  | 7,8-dihydro-8-oxoguanine triphosphatase |  |
| PSMB4 | P28070 |  | Proteasome subunit beta type-4 |  |
| PDPK1 | O15530-1 |  | 3-phosphoinositide-dependent protein kinase 1 |  |
| GIT2 | Q14161-1 |  | ARF GTPase-activating protein GIT2 |  |
| CPNE2 | Q96FN4 |  | Copine-2 |  |
| DENND5B | Q6ZUT9-2 |  | Isoform 2 of DENN domain-containing protein 5B |  |
| ATP6V1G1 | O75348 |  | V-type proton ATPase subunit G 1 |  |
| CYB5R1 | Q9UHQ9 |  | NADH-cytochrome b5 reductase 1 |  |
| CCNT2 | O60583 |  | Cyclin-T2 |  |
| DAB2IP | Q5VWQ8-1 |  | Disabled homolog 2-interacting protein |  |
| LAMTOR3 | Q9UHA4 |  | ragulator complex protein LAMTOR3 |  |
| COA7 | Q96BR5 |  | Cytochrome c oxidase assembly factor 7 |  |
| NUDT16L1 | Q9BRJ7-2 |  | Isoform 2 of Protein syndesmos |  |
| METTL22 | Q9BUU2-2 |  | Isoform 2 of Methyltransferase-like protein 22 |  |
| LYPLA1 | O75608 |  | Acyl-protein thioesterase 1 |  |
| RETREG2 | Q8NC44 |  | Protein FAM134A |  |
| TTC5 | Q8N0Z6 |  | Tetratricopeptide repeat protein 5 |  |
| KLC4 | Q9NSK0-3 |  | Isoform 3 of Kinesin light chain 4 |  |
| PEX13 | Q92968 |  | Peroxisomal membrane protein pex13 |  |
| C7ORF50 | Q9BRJ6 |  | Uncharacterized protein C7orf50 |  |
| PTPN7 | P35236-3 |  | Isoform 3 of Tyrosine-protein phosphatase non-receptor type 7 |  |
| KCTD10 | Q9H3F6-1 |  | BTB/POZ domain-containing adapter for CUL3-mediated RhoA degradation protein 3 |  |
| CCDC85B | Q15834 |  | Coiled-coil domain-containing protein 85B |  |
| TRMT44 | Q8IYL2 |  | probable tRNA (uracil-O(2)-)-methyltransferase |  |
| NUDT8 | Q8WV74 |  | Nucleoside diphosphate-linked moiety X motif 8 |  |
| COMTD1 | Q86VU5 |  | Catechol O-methyltransferase domain-containing protein 1 |  |
| TRUB2 | O95900 |  | Probable tRNA pseudouridine synthase 2 |  |
| ADSS | P30520 |  | adenylosuccinate synthetase isozyme 2 |  |
| C8ORF82 | Q6P1X6 |  | UPF0598 protein C8orf82 |  |
| UQCRB | P14927-2 |  | Isoform 2 of Cytochrome b-c1 complex subunit 7 |  |
| DIABLO | Q9NR28-1 |  | Diablo homolog, mitochondrial |  |
| RCE1 | Q9Y256 |  | CAAX prenyl protease 2 |  |
| FAM76B | Q5HYJ3-1 |  | Protein FAM76B |  |
| LRWD1 | Q9UFC0 |  | Leucine-rich repeat and WD repeat-containing protein 1 |  |
| AIP | O00170 |  | AH receptor-interacting protein |  |
| SCPEP1 | Q9HB40 |  | Retinoid-inducible serine carboxypeptidase |  |
| MAP2K4 | P45985-2 |  | Isoform 2 of Dual specificity mitogen-activated protein kinase kinase 4 |  |
| WBP11 | Q9Y2W2 |  | WW domain-binding protein 11 |  |
| NAT1 | P18440 |  | Arylamine N-acetyltransferase 1 |  |
| UBIAD1 | Q9Y5Z9-1 |  | UbiA prenyltransferase domain-containing protein 1 |  |
| CHAMP1 | Q96JM3 |  | Chromosome alignment-maintaining phosphoprotein 1 |  |
| KIN | O60870 |  | DNA/RNA-binding protein KIN17 |  |
| ATP5MD | Q96IX5 |  | Up-regulated during skeletal muscle growth protein 5 |  |
| THAP11 | Q96EK4 |  | THAP domain-containing protein 11 |  |
| BRK1 | Q8WUW1-2 |  | Isoform 2 of Protein BRICK1 |  |
| LPXN | O60711-2 |  | Isoform 2 of Leupaxin |  |
| TRIP4 | Q15650 |  | Activating signal cointegrator 1 |  |
| TIPRL | O75663 |  | TIP41-like protein |  |
| ZNF519 | Q8TB69 |  | Zinc finger protein 519 |  |
| UBE2C | O00762 |  | Ubiquitin-conjugating enzyme E2 C |  |
| UBE2L3 | P68036-3 |  | Isoform 3 of Ubiquitin-conjugating enzyme E2 L3 |  |
| DCK | P27707 |  | deoxycytidine kinase |  |
| VAT1 | Q99536 |  | Synaptic vesicle membrane protein VAT-1 homolog |  |
| ATPAF1 | Q5TC12-1 |  | ATP synthase mitochondrial F1 complex assembly factor 1 |  |
| BAIAP2 | Q9UQB8 |  | Brain-specific angiogenesis inhibitor 1-associated protein 2 |  |
| NEMP1 | O14524-1 |  | Nuclear envelope integral membrane protein 1 |  |
| SMIM12 | Q96EX1 |  | small integral membrane protein 12 |  |
| LSM11 | P83369 |  | U7 snRNA-associated Sm-like protein LSm11 |  |
| CALB2 | P22676 |  | Calretinin |  |
| TBC1D14 | Q9P2M4-1 |  | TBC1 domain family member 14 |  |
| SCARA3 | Q6AZY7-1 |  | Scavenger receptor class A member 3 |  |
| LRRC42 | Q9Y546 |  | Leucine-rich repeat-containing protein 42 |  |
| RPL36 | Q9Y3U8 |  | 60S ribosomal protein L36 |  |
| PPM1B | O75688 |  | Protein phosphatase 1B |  |
| RHOB | P62745 |  | Rho-related GTP-binding protein RhoB |  |
| DIAPH2 | O60879 |  | Protein diaphanous homolog 2 |  |
| HAUS1 | Q96CS2-1 |  | HAUS augmin-like complex subunit 1 |  |
| TBC1D7 | Q9P0N9 |  | TBC1 domain family member 7 |  |
| NUP210L | Q5VU65 |  | nuclear pore membrane glycoprotein 210-like |  |
| GTF2I | P78347 |  | General transcription factor II-I |  |
| SEPT2 | Q15019-2 |  | Isoform 2 of Septin-2 |  |
| NDUFC2 | O95298-1 |  | NADH dehydrogenase [ubiquinone] 1 subunit C2 |  |
| SUN1 | O94901-9 |  | Isoform 9 of SUN domain-containing protein 1 |  |
| SLC25A51 | Q9H1U9 |  | Solute carrier family 25 member 51 |  |
| STAU1 | O95793 |  | double-stranded RNA-binding protein Staufen homolog 1 |  |
| ZYX | Q15942 |  | Zyxin |  |
| SLC4A7 | Q9Y6M7-7 |  | Isoform 7 of Sodium bicarbonate cotransporter 3 |  |
| QSER1 | Q2KHR3-1 |  | Glutamine and serine-rich protein 1 |  |
| CTSD | P07339 |  | Cathepsin D |  |
| CNOT3 | O75175 |  | CCR4-NOT transcription complex subunit 3 |  |
| RASSF7 | Q02833-1 |  | Ras association domain-containing protein 7 |  |
| MRPS17 | Q9Y2R5 |  | 28S ribosomal protein S17, mitochondrial |  |
| KNL1 | Q8NG31-1 |  | Protein CASC5 |  |
| COPS2 | P61201-2 |  | Isoform 2 of COP9 signalosome complex subunit 2 |  |
| GRSF1 | Q12849 |  | G-rich sequence factor 1 |  |
| ZBTB1 | Q9Y2K1 |  | Zinc finger and BTB domain-containing protein 1 |  |
| MGAT4B | Q9UQ53-1 |  | Alpha-1,3-mannosyl-glycoprotein 4-beta-N-acetylglucosaminyltransferase B |  |
| TVP23C | Q96ET8-1 |  | Golgi apparatus membrane protein TVP23 homolog C |  |
| MRPS31 | Q92665 |  | 28S ribosomal protein S31, mitochondrial |  |
| APC | P25054-1 |  | Adenomatous polyposis coli protein |  |
| RBX1 | P62877 |  | E3 ubiquitin-protein ligase RBX1 |  |
| ZBTB2 | Q8N680 |  | Zinc finger and BTB domain-containing protein 2 |  |
| ECM1 | Q16610-4 |  | Isoform 4 of Extracellular matrix protein 1 |  |
| NAGA | P17050 |  | alpha-N-acetylgalactosaminidase |  |
| ITGB1BP1 | O14713-1 |  | Integrin beta-1-binding protein 1 |  |
| DCAF10 | Q5QP82 |  | DDB1- and CUL4-associated factor 10 |  |
| SLC27A2 | O14975 |  | very long-chain acyl-CoA synthetase |  |
| RPS29 | P62273-1 |  | 40S ribosomal protein S29 |  |
| RRP1B | Q14684 |  | Ribosomal RNA processing protein 1 homolog B |  |
| JPH1 | Q9HDC5 |  | junctophilin-1 |  |
| CHCHD4 | Q8N4Q1-2 |  | Isoform 2 of Mitochondrial intermembrane space import and assembly protein 40 |  |
| MRPL41 | Q8IXM3 |  | 39S ribosomal protein L41, mitochondrial |  |
| ATF6B | Q99941-1 |  | Cyclic AMP-dependent transcription factor ATF-6 beta |  |
| GLO1 | Q04760-1 |  | lactoylglutathione lyase |  |
| FADD | Q13158 |  | FAS-associated death domain protein |  |
| VPS37A | Q8NEZ2 |  | Vacuolar protein sorting-associated protein 37A |  |
| CMBL | Q96DG6 |  | Carboxymethylenebutenolidase homolog |  |
| UBL5 | Q9BZL1 |  | ubiquitin-like protein 5 |  |
| CDC42EP1 | Q00587-1 |  | Cdc42 effector protein 1 |  |
| SMDT1 | Q9H4I9 |  | Essential MCU regulator, mitochondrial |  |
| C2CD2L | O14523-2 |  | Isoform 2 of C2 domain-containing protein 2-like |  |
| DPH6 | Q7L8W6 |  | Diphthine--ammonia ligase |  |
| TNK1 | Q13470-1 |  | Non-receptor tyrosine-protein kinase TNK1 |  |
| FAM50A | Q14320 |  | Protein FAM50A |  |
| IFI44 | Q8TCB0 |  | Interferon-induced protein 44 |  |
| PPP4R4 | Q6NUP7-1 |  | Serine/threonine-protein phosphatase 4 regulatory subunit 4 |  |
| WDR7 | Q9Y4E6-1 |  | WD repeat-containing protein 7 |  |
| PPP1R18 | Q6NYC8 |  | Phostensin |  |
| CHMP1B | Q7LBR1 |  | Charged multivesicular body protein 1b |  |
| MRPS15 | P82914 |  | 28S ribosomal protein S15, mitochondrial |  |
| PEMT | Q9UBM1-2 |  | Isoform 2 of Phosphatidylethanolamine N-methyltransferase |  |
| MRPL50 | Q8N5N7 |  | 39S ribosomal protein L50, mitochondrial |  |
| FKBP15 | Q5T1M5 |  | FK506-binding protein 15 |  |
| SLC22A8 | Q8TCC7-2 |  | Isoform 2 of Solute carrier family 22 member 8 |  |
| AP4E1 | Q9UPM8-1 |  | AP-4 complex subunit epsilon-1 |  |
| CHCHD3 | Q9NX63 |  | MICOS complex subunit MIC19 |  |
| SUB1 | P53999 |  | Activated RNA polymerase II transcriptional coactivator p15 |  |
| PPP2R3B | Q9Y5P8-1 |  | Serine/threonine-protein phosphatase 2A regulatory subunit B'' subunit beta |  |
| RNF157 | Q96PX1 |  | RING finger protein 157 |  |
| TRIM27 | P14373 |  | Zinc finger protein RFP |  |
| FAM53C | Q9NYF3 |  | protein FAM53C |  |
| COTL1 | Q14019 |  | coactosin-like protein |  |
| TRANK1 | O15050 |  | TPR and ankyrin repeat-containing protein 1 |  |
| DTWD1 | Q8N5C7-1 |  | DTW domain-containing protein 1 |  |
| MAP2K5 | Q13163-1 |  | Dual specificity mitogen-activated protein kinase kinase 5 |  |
| CHRNA9 | Q9UGM1 |  | Neuronal acetylcholine receptor subunit alpha-9 |  |
| UQCC3 | Q6UW78 |  | Ubiquinol-cytochrome-c reductase complex assembly factor 3 |  |
| GINS2 | Q9Y248 |  | Dna replication complex gins protein psf2 |  |
| ZFYVE21 | Q9BQ24-2 |  | Isoform 2 of Zinc finger FYVE domain-containing protein 21 |  |
| NDUFB1 | O75438-2 |  | Isoform 2 of NADH dehydrogenase [ubiquinone] 1 beta subcomplex subunit 1 |  |
| PLXND1 | Q9Y4D7-1 |  | plexin-D1 |  |
| IRF2BP1 | Q8IU81 |  | Interferon regulatory factor 2-binding protein 1 |  |
| PIP4K2B | P78356 |  | Phosphatidylinositol 5-phosphate 4-kinase type-2 beta |  |
| SVIL | O95425 |  | Supervillin |  |
| TSEN2 | Q8NCE0-1 |  | tRNA-splicing endonuclease subunit Sen2 |  |
| KIAA0930 | Q6ICG6-2 |  | Isoform 2 of Uncharacterized protein KIAA0930 |  |
| TXNIP | Q9H3M7 |  | thioredoxin-interacting protein |  |
| UBTD2 | Q8WUN7 |  | Ubiquitin domain-containing protein 2 |  |
| VSNL1 | P62760 |  | Visinin-like protein 1 |  |
| KCTD15 | Q96SI1-1 |  | BTB/POZ domain-containing protein KCTD15 |  |
| NUSAP1 | Q9BXS6 |  | Nucleolar and spindle-associated protein 1 |  |
| RHPN1 | Q8TCX5 |  | Rhophilin-1 |  |
| MRM3 | Q9HC36 |  | rRNA methyltransferase 3, mitochondrial |  |
| SURF2 | Q15527 |  | Surfeit locus protein 2 |  |
| MALSU1 | Q96EH3 |  | mitochondrial assembly of ribosomal large subunit protein 1 |  |
| RRN3P2 | A6NIE6 |  | Putative RRN3-like protein RRN3P2 |  |
| ZPR1 | O75312 |  | Zinc finger protein ZPR1 |  |
| EZH2 | Q15910-2 |  | Isoform 2 of Histone-lysine N-methyltransferase EZH2 |  |
| C12ORF40 | Q86WS4 |  | Uncharacterized protein C12orf40 |  |
| TXLNG | Q9NUQ3-1 |  | Gamma-taxilin |  |
| BCR | P11274-1 |  | Breakpoint cluster region protein |  |
| GOLGA1 | Q92805 |  | Golgin subfamily A member 1 |  |
| MGA | Q8IWI9-4 |  | Isoform 4 of MAX gene-associated protein |  |
| PTPRA | P18433 |  | Receptor-type tyrosine-protein phosphatase alpha |  |
| SRRM2 | Q9UQ35 |  | serine/arginine repetitive matrix protein 2 |  |
| MCUB | Q9NWR8 |  | Calcium uniporter regulatory subunit MCUb, mitochondrial |  |
| PCGF6 | Q9BYE7-1 |  | Polycomb group RING finger protein 6 |  |
| LRRC46 | Q96FV0 |  | Leucine-rich repeat-containing protein 46 |  |
| RNF5 | Q99942 |  | E3 ubiquitin-protein ligase RNF5 |  |
| MED14 | O60244 |  | Mediator of RNA polymerase II transcription subunit 14 |  |
| YAF2 | Q8IY57-5 |  | Isoform 2 of YY1-associated factor 2 |  |
| KMT2E | Q8IZD2-1 |  | Histone-lysine N-methyltransferase 2E |  |
| SAMD4A | Q9UPU9-1 |  | Protein Smaug homolog 1 |  |
| FAHD2B | Q6P2I3 |  | Fumarylacetoacetate hydrolase domain-containing protein 2B |  |
| SPC24 | Q8NBT2 |  | kinetochore protein SPC24 |  |
| KRIT1 | O00522 |  | Krev interaction trapped protein 1 |  |
| RTN4IP1 | Q8WWV3-1 |  | Reticulon-4-interacting protein 1, mitochondrial |  |
| RBM10 | P98175-1 |  | RNA-binding protein 10 |  |
| ACVR1 | Q04771 |  | Activin receptor type-1 |  |
| SPSB3 | Q6PJ21 |  | SPRY domain-containing SOCS box protein 3 |  |
| PSMG4 | Q5JS54-2 |  | Isoform 2 of Proteasome assembly chaperone 4 |  |
