# Supplementary material for: IPO5 promotes the proliferation and tumourigenicity of colorectal cancer cells by mediating RASAL2 nuclear transportation
Source: J Exp Clin Cancer Res. 2019 Jul 9;38:296. doi: 10.1186/s13046-019-1290-0 (PMC6617704; doi:10.1186/s13046-019-1290-0)
Supplement: Supplementary file 4 — Table S2. Pathway analysis of IPO5 binding candidates using DAVID tool. (DOCX 16 kb) [file 13046_2019_1290_MOESM4_ESM.docx]

**Table S2. Pathway analysis of IPO5 binding candidates using DAVID tool.**

| Category | Term | Count | PValue |
| --- | --- | --- | --- |
| KEGG_PATHWAY | Ribosome | 15 | 1.99E-04 |
| KEGG_PATHWAY | Spliceosome | 13 | 0.0018197 |
| KEGG_PATHWAY | Ubiquitin mediated proteolysis | 13 | 0.0023399 |
| KEGG_PATHWAY | Regulation of actin cytoskeleton | 15 | 0.0120999 |
| KEGG_PATHWAY | Phagosome | 12 | 0.0130143 |
| KEGG_PATHWAY | Alzheimer's disease | 12 | 0.0277778 |
| KEGG_PATHWAY | Protein processing in endoplasmic reticulum | 12 | 0.0288529 |
| KEGG_PATHWAY | Sphingolipid signaling pathway | 9 | 0.0514018 |
| KEGG_PATHWAY | Parkinson's disease | 10 | 0.0524835 |
| KEGG_PATHWAY | Lysosome | 9 | 0.0535013 |
| KEGG_PATHWAY | Cell cycle | 9 | 0.0601272 |
| KEGG_PATHWAY | Huntington's disease | 12 | 0.0625067 |
| KEGG_PATHWAY | Axon guidance | 9 | 0.0672494 |
| KEGG_PATHWAY | Influenza A | 11 | 0.0723575 |
| KEGG_PATHWAY | Ribosome biogenesis in eukaryotes | 7 | 0.0751403 |
| KEGG_PATHWAY | Proteoglycans in cancer | 12 | 0.0771152 |
| KEGG_PATHWAY | Hepatitis C | 9 | 0.082998 |
| KEGG_PATHWAY | Oxidative phosphorylation | 9 | 0.082998 |
| KEGG_PATHWAY | Amyotrophic lateral sclerosis (ALS) | 5 | 0.0891958 |
| KEGG_PATHWAY | Inositol phosphate metabolism | 6 | 0.0932296 |
| KEGG_PATHWAY | Herpes simplex infection | 11 | 0.0937354 |
| KEGG_PATHWAY | Vibrio cholerae infection | 5 | 0.0996206 |
