# Supplementary material for: IPO5 promotes the proliferation and tumourigenicity of colorectal cancer cells by mediating RASAL2 nuclear transportation
Source: J Exp Clin Cancer Res. 2019 Jul 9;38:296. doi: 10.1186/s13046-019-1290-0 (PMC6617704; doi:10.1186/s13046-019-1290-0)
Supplement: Supplementary file 5 — Table S3. List of 15 IPO5 binding proteins with NLS sequence. (DOCX 20 kb) [file 13046_2019_1290_MOESM5_ESM.docx]

**Table S3. List of 15 IPO5 binding proteins with NLS sequence**

|  | **UniProt ID** | **Signal** | **SignalType** | **Start** | **End** |
| --- | --- | --- | --- | --- | --- |
| UBR5 | O95071 | TIKKK | NLS | 859 | 863 |
| DNAJB1 | P25685 | RRKVP | NLS | 294 | 298 |
| ATRX | P46100 | GKRKRK | NLS | 777 | 782 |
| ATRX | P46100 | KKKKRD | NLS | 2222 | 2227 |
| ATRX | P46100 | KPKEH | NLS | 1359 | 1363 |
| ATRX | P46100 | KRKRKS | NLS | 778 | 783 |
| ATRX | P46100 | RKRQD | NLS | 1102 | 1106 |
| LIMK1 | P53667 | KKPDR | NLS | 499 | 503 |
| RAD51 | Q06609 | RLADEF | NES | 254 | 259 |
| ORC2 | Q13416 | KRAKLD | NLS | 263 | 268 |
| ORC2 | Q13416 | KRMKR | NLS | 229 | 233 |
| RABL6 | Q3YEC7 | KKKSKH | NLS | 670 | 675 |
| RABL6 | Q3YEC7 | KSKHKK | NLS | 672 | 677 |
| ZMYM4 | Q5VZL5 | PRRRGR | NLS | 1173 | 1178 |
| ZMYM4 | Q5VZL5 | RRGRKK | NLS | 1175 | 1180 |
| ZMYM4 | Q5VZL5 | SRTRRR | NLS | 1160 | 1165 |
| SIN3A | Q96ST3 | KRRLDD | NLS | 2 | 7 |
| SIN3A | Q96ST3 | QIRRH | NLS | 426 | 430 |
| BRWD1 | Q9NSI6 | KRRKTK | NLS | 2177 | 2182 |
| BRWD1 | Q9NSI6 | KRRRKR | NLS | 908 | 913 |
| BRWD1 | Q9NSI6 | PKRRRK | NLS | 907 | 912 |
| BRWD1 | Q9NSI6 | RKTKGK | NLS | 2179 | 2184 |
| BRWD1 | Q9NSI6 | RRKRNR | NLS | 1514 | 1519 |
| BRWD1 | Q9NSI6 | RTCRR | NLS | 796 | 800 |
| BRWD1 | Q9NSI6 | SPPKRR | NLS | 905 | 910 |
| STAU2 | Q9NUL3 | KKLPPLPVVEKPKLFFKKR | NLS | 273 | 291 |
| PRKAG2 | Q9UGJ0 | THKRI | NLS | 400 | 404 |
| RASAL2 | Q9UJF2 | KKKKKDK | NLS | 237 | 243 |
| ZC3H4 | Q9UPT8 | RLKRKR | NLS | 102 | 107 |
| ZC3H4 | Q9UPT8 | SKKRRK | NLS | 118 | 123 |
| RRP7A | Q9Y3A4 | RRKRSR | NLS | 230 | 235 |
