# Supplementary material for: IPO5 promotes the proliferation and tumourigenicity of colorectal cancer cells by mediating RASAL2 nuclear transportation
Source: J Exp Clin Cancer Res. 2019 Jul 9;38:296. doi: 10.1186/s13046-019-1290-0 (PMC6617704; doi:10.1186/s13046-019-1290-0)
Supplement: Supplementary file 6 — Table S4. Primer sequences used in RT-qPCR analysis. Table S5: Nucleotide sequences used for knockdown. (DOC 40 kb) [file 13046_2019_1290_MOESM6_ESM.doc]

**Table S4. Primer sequences used in RT-**qPCR analysis.

| Target | | Forward primer (5’-3’) | | Reverse primer (5’-3’) | |
| --- | --- | --- | --- | --- | --- |
| IPO5 | CTGCTGAAGAGGCTAGACAAATG | | TCTGCCGCAATATCACAAACTT | |  |
| GAPDH | GGAGCGAGATCCCTCCAAAAT | | GGCTGTTGTCATACTTCTCATGG | |  |

**Table S5. Nucleotide sequences used for** knockdown.

|  | target sequence (5’-3’) |
| --- | --- |
| IPO5 siRNA-1 | TGCCGATGATCAAGGAACA |
| IPO5 siRNA-2 | AGGCGAGAGTGCTCTAGAT |
